# Glycerosomal thermosensitive in situ gel of duloxetine HCl as a novel nanoplatform for rectal delivery: in vitro optimization and in vivo appraisal

**DOI:** 10.1007/s13346-022-01172-z

**Published:** 2022-05-27

**Authors:** Heba F. Salem, Adel A. Ali, Yasmine K. Rabea, Fatma I. Abo El-Ela, Rasha A. Khallaf

**Affiliations:** 1 Department of Pharmaceutics and Industrial Pharmacy, Faculty of Pharmacy, Beni-Suef University, Beni-Suef, Egypt; 2 Department of Pharmacology, Faculty of Veterinary Medicine, Beni-Suef University, Egypt, 62511 Egypt

**Keywords:** Duloxetine HCl, Depression, Glycerosomes, Rectal delivery, Pharmacokinetics, Pharmacodynamics, Thermosensitive in situ gel

## Abstract

Duloxetine HCl (DXH) is a reuptake inhibitor of serotonin and norepinephrine used to treat the major depressive disorder. Following its extensive hepatic metabolism, acid-labile nature, and limited aqueous solubility, DXH has poor oral bioavailability (40%). The rectal route has been suggested as another route of administration to surmount such challenges. The present study aimed to prepare DXH-loaded glycerosomal (DXH-GLYS) in situ gel for rectal administration to increase DXH permeability and improve its bioavailability. Box–Behnken design (BBD) was adopted to prepare and optimize nanoglycerosomes. The impact of Phospholipon 90G (PL90G), Tween 80 concentrations, and glycerol percentage on encapsulation efficiency, nanoglycerosomal size, % cumulative DXH released, and the cumulative DXH permeated per unit area after 24 h were studied by the design. The pharmacokinetic and pharmacodynamic behavior of optimized formulation was investigated in rats. The formulated DXH-GLYS had a vesicle size ranging between 135.9 and 430.6 nm and an entrapment efficiency between 69.11 and 98.12%. The permeation experiment revealed that the optimized DXH-GLYS in situ gel increased DXH permeation by 2.62-fold compared to DXH solution. Pharmacokinetics studies disclosed that the DXH-GLYS in situ rectal gel exhibited 2.24-times increment in DXH bioavailability relative to oral DXH solution. The pharmacodynamic study revealed that the DXH-GLYS rectal treatment significantly improved the behavioral analysis parameters and was more efficacious as an antidepressant than the oral DXH solution. Collectively, these findings demonstrate that GLYS can be considered a potentially valuable rectal nanocarrier that could boost the DXH efficacy.

**Graphical abstract:**

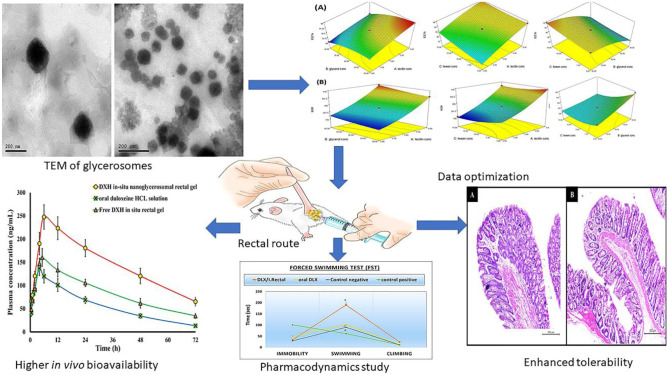

**Supplementary Information:**

The online version contains supplementary material available at 10.1007/s13346-022-01172-z.

## Introduction

Duloxetine HCl (DXH), a dual serotonin and norepinephrine reuptake inhibitor, has a chemical name of (3S)-N-methyl-3-naphthalen-1-yloxy-3-thiophen-2ylpropan-1-amine. In 2002, it was approved by the United States FDA for the management of depression, generalised anxiety disorder [1], female stress urinary incontinency, fibromyalgia, and peripheral neuropathy [2]. Compared with other antidepressants, it has favorable pharmacodynamic characteristics, such as dual inhibition, tolerance and safety, faster recovery, fewer adverse effects, and a low possibility of binding with any other neural receptors [3–5].

DXH is well absorbed when administered orally; nonetheless, it takes two hours as the lag time before absorption begins, and six hours after dosing till reaching its t_max_. The oral bioavailability of DXH is low and fluctuates from 30 to 60%. Such poor bioavailability could be ascribed to excessive 1^st^-pass metabolism via cytochrome P450 P1A2 and limited solubility since it belongs to BCS class II drugs. DXH is also easily degraded in the acidic gastrointestinal (GIT) medium, resulting in low therapeutic levels and diminished clinical efficacy [6, 7]. Additionally, the conventional DXH multiple regular doses or a multimodal treatment strategy involving the administration of two or more drugs from different therapeutic antidepressant classes are mandatory to avoid patients suffering from suboptimal relief, leading to poor patient compliance. Considering that, a rectal delivery system is required as an alternative to the conventional DXH dosage forms.

The rectal drug administration system is generally deemed as a noninvasive, locally targeted system that can minimize systemic toxicity. With the recently developed drug delivery techniques, it is also being considered for systemic drug administration. Rectal route has several advantages, including less vulnerability to enzymatic degradation alongside its aptitude to partially curtail the drugs’ presystemic metabolism, where the lower part of the rectal venous blood flow is directly connected to the systemic circulation. As a result, a more stable environment for drug absorption was provided, allowing the plasma drug concentration to be in a constant and steady-state, reducing the drug administration intervals and improving patient compliance [8, 9].

Patient discomfort and rejection are common side effects of traditional suppositories which impair patient compliance [10]. Additionally, if they lack adherence to the mucous membranes, such conventional dosages can easily reach the colon’s end, exposing the laded drugs to first-pass metabolism [11–13]. Consequently, another easy-to-administer rectal formulation with suitable bioadhesive strength must be used [14]. The thermosensitive mucoadhesive in situ gel, which exists as liquid at ambient temperature and transforms into a gel state inside the body, was exploited to extend the preparation's residence time and thereby boost its absorption rate. This gel-type is widely studied for nasal, intraperitoneal, subcutaneous, topical, ocular, and rectal delivery systems [15, 16]. Poly(*N*-isopropyl acrylamide), poloxamers (Pluronic^®^), and cellulose derivatives are the commonly utilized thermosensitive polymers in drug delivery.

Phospholipid nano-vesicular systems have aroused great interest, especially as a promising drug delivery system, which can enhance the transdermal, dermal, and transmucosal absorption of numerous drugs and evade their degradation in GIT and liver [17]. Liposomes were the first generation of phospholipid-based vesicles, comprising one or more lipidic bilayers around an aqueous core, allowing them to encapsulate a wide variety of drug molecules, either hydrophilic or lipophilic [18]. Over the past decades, various additives like ethanol and surfactants had been used to alter the physical, chemical, and functional properties of liposomes, and newer generations (known as elastic or ultra-deformable liposomes) have been invented, allowing for more efficient drug delivery across the biological membranes [19–22]. Invasomes, ethosomes, transferosomes, and glycerosomes are examples of these innovative systems that have been extensively investigated for dermal, transdermal, transmucosal, and rectal administration of several drugs [23–26].

Glycerosomes (GLYS) were first introduced as novel flexible vesicular nano-cargos in 2012, prepared from dipalmitoylphosphatidylcholine as a backbone and different percentages of harmless glycerol, up to 40% in the aqueous phase [27]. The integration of glycerol in high quantities enhances the permeability of GLYS, pursuant to its ability to modify the phospholipid bilayer fluidity, with further amelioration of glycerosomal penetration and absorption. GLYS might contain additives like cholesterol, which enhance PL90G bilayer stability, and they could also be produced employing the same methods used in a conventional liposome preparation.

Recently, GLYS have been proposed for topical, intranasal, and transdermal drug delivery as well as inhalable delivery systems [28–31]. Indeed, various trials have been carried out to improve DXH bioavailability, including intranasal DXH-proniosomal thiomer gel and intranasal DXH-cubosomal in situ gel for enhancing DXH bioavailability and its brain targeting potential [32, 33]. Until now, no trials have been explored utilizing GLYS to increase DXH bioavailability. Moreover, the literature lacks any data on the capability of GLYS to improve the transmucosal permeability of rectally administrated drugs. Consequently, current study is concerned with tailoring and appraisal of novel nanovesicle in situ gelling system as a suitable paradigm for the rectal administration of DXH, addressing the issues of DXH oral delivery. In this perspective, the fabricated GLYS are physio-chemically characterizing adopting the BBD, and the optimal glycerosomal dispersion was then integrated into an in situ gel base that was evaluated in vitro and in vivo.

## Materials and methods

### Materials

Duloxetine HCL (DXH) was obtained by EVA pharma (Cairo, Egypt) as a gifted sample, and Phospholipon 90G (PL90G) was donated from lipoid GmbH (Nattermannallee, Germany). Glycerol, Cholesterol, Tween 80, Poloxamer 407 (P407), Poloxamer 188 (P188), hydroxypropyl methylcellulose (HPMC, K15M), and acetonitrile (HPLC grade) were procured from Sigma-Aldrich (St. Louis, MO.USA). All other chemicals and solvents used were of analytical grade. Double deionized water was used throughout all the research.

### Experimental design

The causal factors and their coded levels (low, medium, and high) were chosen in this study based on literature review data and previous screening trials. The independent variables elected for optimization were PL90G concentrations (2, 3, and 4%w/v) (A), glycerol percentages (20, 30, and 40% (v/v) (B), and surfactant concentrations (5, 10 and 15% w/w from the total lipids used) (C). Entrapment efficiency (EE %, Y_1_), nanoglycerosomal size (Y_2_), cumulative DXH released (%CDXR, Y_3_), and the cumulative DXH permeated per unit area after 24 Q_24_ (μg/cm^2^) (Y_4_) were selected as the dependent responses. Fifteen runs were prepared using the three-level, three-factor (3^3^) Box–Behnken design created with Design-Expert^®^ software (version 10.0.0.3, Manugistics Inc., Rockville, USA). Table 1 displayed the compositions of the developed GLYS along with their observed responses. The best suitable model (linear, two-factors interaction, or quadratic model) for each response was chosen according to the resulted statistical parameters. The polynomial equations that represent each model were also obtained and defined as:

**Table 1:** Box-Behnken design responses of DXH nano-glycerosmes formulations

**Formulation code**	**Independent variables**			**Dependent variables (Mean ± SD)**				Poly dispersity
	**A**	**B**	**C**	**Y**_**1**_	**Y**_**2**_	Y_3_	Y_4_	Index (PDI)
**1**	1	1	0	80.22 ± 0.95	430.6 ± 3.9	34.08 ± 3.00	445.55 ± 11.00	0.621
**2**	-1	0	-1	75.38 ± 0.82	200.2 ± 5.67	55.23 ± 1.30	± 522.21 ± 9.03	0.694
**3**	-1	1	0	69.11 ± 0.83	160.9 ± 2.97	41.17 ± 1.42	540.07 ± 19.57	0.475
**4**	-1	0	1	83.76 ± 0.32	140.0 ± 1.25	54.81 ± 1.03	527.37 ± 10.21	0.348
**5**	-1	-1	0	81.79 ± 0.99	135.9 ± 2.32	60.52 ± 1.51	486.21 ± 9.62	0.439
**6**	0	1	-1	73.54 ± 0.92	333.5 ± 4.31	42.23 ± 3.8	385.61 ± 12.65	0.707
**7**	0	0	0	86.21 ± 0.56	234.8 ± 9.72	50.64 ± 2.21	440.22 ± 3.68	0.401
**8**	0	0	0	87.76 ± 0.54	250.8 ± 2.01	52.05 ± 0.98	427.45 ± 8.54	0.450
**9**	1	0	-1	93.21 ± 0.13	424.9 ± 5.52	30.96 ± 2.5	332.5 ± 17.99	0.635
**10**	0	-1	1	96.01 ± 0.81	226.3 ± 5.00	58.83 ± 0.89	370.45 ± 11.84	0.287
**11**	1	-1	0	98.12 ± 0.73	375.7 ± 2.0	37.94 ± 1.41	320.23 ± 7.00	0.690
**12**	0	0	0	86.99 ± 0.33	240.3 ± 5.0	52.37 ± 0.72	429.43 ± 6.8	0.248
**13**	1	0	1	94.65 ± 0.89	395.5 ± 2.32	40.68 ± 1.49	405.6 ± 9.85	0.581
**14**	0	1	1	32.77 ± 0.44	236.9 ± 1.58	43.96 ± 1.20	500.32 ± 15.29	0.513
**15**	0	-1	-1	89.87 ± 0.29	259.6 ± 3.21	56.52 ± 1.36	365.3 ± 2.62	0.370

N.B. A: Phospholipon 90G (PL90G), B: glycerol percentages, C: tween 80 concentrations\

Y_1_: EE%, Y_2_: nano-glycerosomal size, Y_3_: %CDXR, and Y_4_: Q_24_ (μg/cm^2^)

1 $$\begin{aligned}\mathrm R=&\;{\mathrm b}_0+\mathrm b1\mathrm A+{\mathrm b}_2\mathrm B+{\mathrm b}_3\mathrm C+{\mathrm b}_{12}\mathrm{AB}+{\mathrm b}_{13}\mathrm{AC}+{\mathrm b}_{23}\mathrm{BC}\\&+{\mathrm b}_{11}\mathrm A^2+{\mathrm b}_{22}\mathrm B^2+{\mathrm b}_{33}\mathrm C^2\end{aligned}$$

where, R and b_0_ are the selected response and the intercept, respectively. The defined factors that have been studied are A, B, and C, and the regression coefficients calculated based on the R values are b_1_ to b_33_. The interaction coefficients are AB, AC and BC, whereas A^2^, B^2^ and C^2^ are the quadratic terms. Observed responses were statistically analyzed using analysis of variance (ANOVA) to estimate the significance of every model at *p* ≤ 0.05. Interaction and 3-dimensional plots (3-D) were created for the four responses to study the interactions among the causal factors.

### Fabrication of DXH-GLYS

DXH-GLYS were developed following the thin film hydration technique. Accurately weighed quantities of PL90G, Tween 80, cholesterol, and DXH were all dissolved in 10 ml of chloroform in a round bottom flask. Fixed amounts of cholesterol and DXH were used in all formulations (20 mg and 10 mg, respectively). The resultant clear organic solution was evaporated using a rotary evaporator (Stuart rotary evaporator, RE300, Wolf temperature laboratories, North Yorkshire, UK, with Stuart vacuum pump, RE3022C, Wolf Laboratories, NorthYorkshire, UK) under vacuum at 60 °C for 30 min to attain a thin homogenous film without any solvent traces on the flask wall. A 10 ml phosphate buffer saline (PBS) (PH 7.4) contained glycerol at different percentages (20, 30, 40% v/v) was used to hydrate the formed thin film. Furthermore, the prepared glycerosomal dispersions were put in a bath sonicator (Sonix TV ss-series ultrasonicator, USA) for 5 min to produce nano-sized suspensions [28].

## Characterization of DXH-GLYS

### Determination of DXH entrapment efficiency

The sonicated nanoglycerosomal suspensions were separated from the non-encapsulated DXH using an exhaustive dialysis method. Each prepared formulation was dialyzed against deionized water (500 ml) using a dialysis membrane (12 to 14 kDa MW cut-off, 3 nm pore size; Spectrum Laboratories Inc., Egypt) at 8 °C for 2 h, which was effective in removing the untrapped DXH [34]. For vesicle disruption, 1 ml of the separated GLYS from each formulation was added to 5 ml of ethanol [35]. After proper dilution with phosphate buffer solution, the DXH quantity was assessed using a UV spectrophotometer (Jasco, V-530, Tokyo, Japan) at *λ*_*max*_ 289. Measurements were accomplished in thrice at different periods. Equation (2) was used to measure the percent DXH entrapped as follows:

2 $$\mathrm{EE{\% }}= \mathrm{Entrapped\ DXH\ /Total\ DXH} *100$$

### Determination of vesicle size

The average diameter of DXH-GLYS and polydispersity index (PDI) were analyzed using a Malvern Zeta Sizer (Malvern, Germany) at 25 ± 2 °C by dynamic light scattering technique (DLS). Prior to the measurement, the freshly prepared nano-suspensions were properly diluted (1:10) using deionized water. The scattering angle was set to 90 degrees [36]. Three determinations were provided for each formulation.

### In vitro release study of DXH from DXH-GLYS

The DXH release from different glycerosomal formulations and DXH solution was performed using a modified Franz diffusion cell with a 6.15 cm^2^ effective diffusion area. The glycerosomal nanosuspension of different formulations (equivalent to 4 mg of DXH) were loaded into the doner part of the Franz cell. Then, it diffused through a dialyzing cellulose membrane (Mw cut-off = 12 000 Da) that had been stored overnight in the releasing vehicle before initiation of the release study. Fifty-milliliters of PBS with pH 7.4 (composition; 8 gm NaCl, 1.44 gm Na_2_HPO_4_, 0.2 gm KCL, 0.24 gm KH_2_PO_4_ dissolved in 1000 ml of distilled water) was placed in the receptor chamber [37]. The receptor chamber was agitated at 100 rpm with the temperature adjusted to be around 37 ± 0.5 °C during the whole dissolution study. Samples of volume 2 ml were taken at regular periods (0.5, 1, 2, 4, 6, 8, and 24 h) from each releasing chamber. The removed volume was immediately substituted using an equal volume of fresh media every time a sample was taken to warrant a constant volume (sink conditions), DXH content was measured spectrophotometrically at *λ*_*max*_ 289 nm in all the withdrawn samples. The release experiment was iterated thrice, and the mean of three readings was computed. The percent accumulative DXH released was expressed using Eq. 2:

3 $$\begin{aligned}&\mathrm{Cumulative\ DXH\ released}\left(\mathrm{\%}\right)=\mathrm{\%\ release\ at\ time}\;\text{"t"}\\&+ \left(\frac{\mathrm{Volume\ at\ sample\ withdrawn}}{\text{release medium volume}}\right)\\&\mathrm{ \%\ released\ previous\ to\; {"t"} }\left(t-1)\right)\end{aligned}$$

DXH release kinetics were determined by fitting the obtained release data to zero-order, first-order, and Higuchi diffusion models. The selection of the model fitting the DXH release mechanism was made based on the model that gave the highest correlation coefficient value (R^2^) [38].

### Ex vivo permeation study

A fabricated Franz diffusion cell with a surface area of 6.15 cm^2^ was used to study the permeation of DXH from the prepared nanoglycerosomal formulations and DXH solution. Freshly excised cattle rectum was used as a permeation model membrane, which was kept in PBS (pH 7.4) for 1 h to equilibrate before the study. PBS (PH 7.4) with a volume of 50 ml was used to fill the receptor chamber, which was continuously agitated at a rate of 100 rpm for 24 h and thermostated at 37 ± 0.5 °C. All the procedures used herein were previously reported by Salem et al. (2017) [14]. In the donor part of the Franz cells, different volumes of DXH-GLYS suspensions (equivalent to 4 mg of DXH) were added to be in direct contact with the rectal mucosa. A 2 ml sample was drawn from the receptor chamber at programmed intervals of 0.5, 1, 2, 4, 6, 8, 12, and 24 h, and then the withdrawing vehicle was quickly substituted with a fresh buffer solution of a similar volume at each time to maintain sink conditions. Filtered and properly diluted withdrawn samples were subjected to spectrophotometric analysis at 289 nm to quantify DXH concentration. The accumulative quantity of DXH permeated across the cattle rectum was calculated for each time interval according to Eq. 3 [28]:

4 $$\mathrm{Cumulative\; amount} = V1 \times Cn + [V2 (\sum C1 + \cdots + Cn-1)]$$

where, V_1_ is the receptor chamber volume (50 ml), V_2_ is the volume taken at each interval (2 ml), and C_n_ is the concentration of the sample that attained at time n.

Triplicate measurements were developed for each formulation (three isolated samples) in the experiment, and the data were expressed as an average ± SD. Cumulative DXH permeated over 24 h per squared cm; Q_24_ (μg /cm^2^) was plotted versus time, and accordingly, all the rectal permeation parameters can be provided. DXH steady-state flux (J_ss_) (μg/cm^2^/h) was derived from the straight line’s slope [39]. The permeability coefficient K_P_ (cm/h) of DXH for all the formulations was calculated by dividing Jss (μg/cm^2^/h) by the initial DXH concentration loaded in the doner compartment [40]. The DXH time to start permeation (lag time in min) was determined from the x-intercept of the linear portion [14, 40].

## Appraisal and optimization of DXH-GLYS

### Experimental model evaluation

Design-expert software was employed to analyze all quantitative outcomes of Y_1_-Y_4_ using the ANOVA test at *p* < 0.05. It provides a model matrix for evaluating the best-fitted model and polynomial equations to assess the mathematical relationships between independent variables. The point prediction method of the Design-Expert software was applied to detect the optimized formulation depending on its ability to establish the independent variable levels (A, B, and C), achieving the smallest glycerosomal size correlated with the greatest EE %, %CDXR, and Q_24_. The opted optimum composition was then exposed for further examination.

### Characterization of the optimized DXH-GLYS formulation

The optimum formulation was prepared and enrolled in many studies, including the measurement of its entrapment efficiency, vesicle size, zeta potential, and stability study. In vitro release behavior and ex vivo diffusion study were also carried out for the optimized formulation employing the same technique and procedures outlined above. Transmission electron microscopy (TEM) (Jeol, Tokyo, Japan) was used to examine the shape and morphology of the optimized formulation. The enhancement ratio (ER) was also determined to assess the nanoglycerosomes’ effectiveness in enhancing DXH permeability compared to DXH solution. ER was computed depending on the equation below:

5 $$\begin{aligned}\mathrm{Enhancement\; ratio}=&\; \mathrm{Jss\ of\ the\ optimized\ DXH-GLYS}\\&/{}\mathrm{ Jss\ of\ the\ DXH\ solution}\end{aligned}$$

### Zeta potential

The zeta potential (ZP) of the optimum nonoglycerosomal dispersion was measured utilizing Malvern Zeta Sizer (Malvern, UK), and the average of triple measurements was computed (n = 3). In ZP measurement, the velocity of glycerosomal vesicles through a liquid was measured employing an electrophoresis-based technique after an electrical field was applied.

### Morphology of nanoglycerosomes

A high-resolution TEM with an operating voltage of 80 kV was used to examine the shape of the optimized DXH-GLYS formulation along with its surface morphology and size. On a carbon-coated cupper array, one drop of the freshly diluted formulation was placed. After applying 1% phosphotungestic acid solution (%w/v) for negative staining, the air-dried samples were photographed [37].

### Stability study

The optimized DXH-GLYS formulation stored in a tightly sealed glass vial was maintained at 4 °C for three months. Collected samples from the optimized formulation after 30, 60, and 90 days were characterized by measuring their zeta potential, vesicle size, as well as EE% and all were compared to the freshly prepared one to check the physical stability of the optimized formulation [41]. All of the obtained data were the average of three measurements ± SD.

#### Preparation of DXH-GLYS thermosensitive in situ gel

For fabricating DXH-GLYS thermally-triggered in situ gel, P407, P188, and HPMC K15M with the percentages of (20:3:0.5%) were used. The cold method described by Schmolka et al. was slightly modified to prepare in situ gel on a weight/volume basis [42]. Briefly, the calculated amount of mucoadhesive polymer)HPMC K15M( was sprinkled onto the freshly prepared optimized DXH-GLYS suspension while stirring continuously till the mixture was totally dissolved. Afterwards, a combination of P407 and P188 was incorporated, properly mixed, and then stored at 4 ºC until needed. The concentration of DXH used was 0.8%. In situ gel formulation of free DXH was also prepared adopting the same method for comparison purposes.

### Characterization of DXH-GLYS in situ gelling system

The freshly fabricated DXH-GLYS in situ gel was evaluated by visual inspection for its organoleptic properties (texture and transparency). In vitro release and ex vivo permeability of the in situ gel were also assessed by the same method previously informed.

### pH measurement

Since the in situ gel preparation was intended for rectal administration, pH evaluation was required to ensure that it was non-irritating. A pH meter was used to measure the pH of the gel in triplicate.

### Spreadability

A simple technique was used to assess the spreadability of the gel. Briefly, 0.5 g of the gelled formulation was placed on a predetermined mark on a glass plate, which was then covered with another plate. The upper plate was compressed for 5 min at constant pressure, with no further spreading expected. The diameter of the spreaded circle around the mark was measured.

### Drug content

0.1 g sample was withdrawn from the in situ gelled formulation and then sonicated using 5 ml of ethanol for vesicle disruption. After proper dilution with PBS solution, the DXH content was analyzed spectrophotometrically at *λ*_*max*_ 289.

### Gelation temperature and time

The method informed by Mansour et al. was used for measuring the sol–to–gel phase transition temperature (gelation temperature) of the DXH-GLYS in situ gel [43]. An aliquot of 2 ml of the in situ gel formulation was transmitted into a test tube concealed using a parafilm and immersed in a thermostatic digital water bath set at 25 °C. The temperature of the water bath was gradually elevated by 1 °C and equilibrated for 10 min with each new adjusted temperature. The thermogel sample was checked for gelation regularly. The gel sample was assessed, in triplicates, for gelation suggested to be occurred once the gel meniscus stopped moving when tilted by 90 degrees [44, 45]. The time of the first gelling detection was defined as gelation time. Three successive measurements were taken.

### Measurement of gel strength

A method described by El Leithy et al. was used to determine the in situ gel strength [9]. In a thermostatically controlled water bath, a sample of 50 g of the optimized DXH-GLYS in situ gel was put in a 100 ml graduated cylinder. The water bath temperature was equilibrated at the predetermined gelation temperature of the DXH-GLYS in situ gel. On the gel surface, a standard weight of 35 g was placed. The time it took the weight to penetrate 5 cm deep into the gelled formulation in sec was measured and considered an arbitrary gel strength index. For rectal application, a time range of 10–50 s was deemed acceptable.

### Gelling capacity

Gelling capacity was determined by examining the gel formulation behaviors, such as gelation and erosion times, as a function of environmental changes. The coding for the gelling capacity is described as reported in literature as follows: + gelled after a few minutes and dissolves rapidly, +  + gelled after a few minutes and remains intact for a few hours, and +  +  + gelled immediately and remains intact for a prolonged time [46, 47].

### In vitro mucoadhesion measurement

A mucoadhesive measuring device constructed in our laboratory was used for evaluating the bioadhesive strength of the DXH-GLYS in situ gel using a modified physical balance. The principle applied was measuring the force necessary to separate the gel preparation from the rectal mucosal tissue. Using cyanoacrylate glue, a segment of freshly excised rectal mucosa from the fundus of the rat was mounted with the mucosal side upwards onto each of the two glass slides. On the first slide, 0.2-g of in situ gel was deposited, and this slide was positioned on a height-adjustable pan. Afterward, another slide with the rectal mucosa was fixed in an inverted manner, attached to the balance. With gel preparation in between, both the slides were kept close to each other for 2 min. The weight continued to rise at the other end of the balance until the detachment of the two slides [48]. The least weight that could detach the rectal mucosal tissue from the thermogel surface was used for calculating the mucoadhesive force (detachment stress in Dynes/cm^2^) as follows:

6 $$\mathrm{Mucoadhesive\ strength\ (Dynes/cm2)} = \mathrm{m *g/a}$$

where, m is the weight needed for separating two slides in grams, g is the acceleration of gravity (980 cm/sec^2^), and A is the area of rectal mucosa.

### Rheological properties determination

Cone and plate viscometer (USA) was used to investigate the rheological properties of the optimized DXH-GLYS thermosensitive in situ gel before and after gelation. At 25 and 37 °C, viscosity measurements were taken using a circulating bath linked to the viscometer over a shear rate range of 20 to 400 (sec^−1^) [48]. The S64 spindle was selected to be dipped in the tested gel. The area of hysteresis loops was also detected by applying a trapezoidal rule.

### In vivo localization and retention of DXH-GLYS thermosensitive in situ gel in the rectum

To examine rectum retention and localization of DXH-GLYS in situ gel formulation, the method described by Choi et al. [13] was implemented using four albino Wistar rats weighing 180–200 g. The optimized DXH-GLYS thermosensitive in situ gel formulation containing 0.1% (w/v) methylene blue was administered into the rectum 4 cm above the anus via a stomach sonde needle fitted to a disposable plastic syringe. Rats were sacrificed 5 min, and 6 h after in situ gel administration, their abdomens were opened and the color of the dye ascertained the localization of the formulation.

## In vivo studies

For the prepared formulation, Pharmacokinetics (PK) and Pharmacodynamics (PD) tests were conducted. This study met the standards of Beni-Suef University animal ethical committee guidelines, the Helsinki protocols, and their approval was sorted before the beginning of the experiment (approval code: BSU-IACUC 021–190). All animal handling procedures complied with the protocol approved by the institutional animal care and use committee of Beni-Suef University's Faculty of Veterinary Medicine, Egypt.

## Pharmacodynamic study

### Animals

The experiment was conducted with 20 adult male albino rats, split into four groups of five animals each. Rats were acclimatized to the basic rodent food for 15 days after their arrival before being subjected to depression induction or the experimental procedures. The rats also had access to clean, fresh water all the day. To avoid defecation throughout the experiment, the rats given the rectal formulation were deprived of any food for 16 h prior to the commencement of receiving the dose, with only water permitted.

### Depression induction and experimental protocol

All animals except the normal control group were forced to swim within a water tube (height: 40 cm; diameter 25 cm( holding 30 cm of water (temperature; 25 ± 2 °C). Following the techniques described by Kaur et al. (2016), rats were subjected to swim sessions every day for 7 days [49]. They underwent a 15-min pre-test swim period on the first day, followed by a 6-min swim period the next day in two sessions, “night and day”. Rats were classified as G1; non-depressed (negative control), whilst G2 was treated with 0.5 ml of normal saline as a positive control depressed rats. G3 was treated with DXH solution in purified water (2.5 mg/ml; 20 mg/kg) orally. G4 was treated with 400 μl of the optimized DXH-GLYS thermosensitive in situ gel formulation containing 4 mg of the drug (20 mg/kg) rectally. Oral dosages were administered using a 16-gauge ball-tipped feeding needle and syringe. For intrarectal dosing, a plastic syringe attached to a stomach sonde needle was used to administer the gel formulation above the anus, at a depth of 4 cm. The rats were hung in a ventral posture with their heads down and the rectum shut for about 10 min until the medication had completely dissolved.

### Forced swimming test (FST)

FST is a rodent behavioral test often used in depression research, especially as a screening tool for acute antidepressants. Immobility is perceived as depression-like behavior (behavioral despair) in this test. The rats were placed inside a barrel filled with water for the restricted or mandatory swimming test. Following a 10-min dosage, the swimming time of the rats was assessed. Antidepressant-treated rats will actively undertake escape-directed behaviors for a more extended time than saline-treated rats [50].

### Tail suspension test

Rats were hung by their tails from a level bar using adhesive tape. The behavior of each rat was assessed 30 min after drug administration for a period of 5 min [51].

### Sucrose preference test

A reduced preference for sweet food in the sucrose inclination test indicates anhedonia, which can be reversed with long-term antidepressant medication. Throughout the training session, from day 1 to day 4, all rats received varied foodstuffs, with two bottles of pure water available on days 1 and 2, two bottles of 1% sucrose on day 3, and one bottle of pure water and one bottle of 1% sucrose on day 4 [52]. Afterward, there were two test sessions after 1 h and again after 12 h of water and food deprivation to all the animals. Each rat was given 200 ml of clean water and 200 ml of 1% sucrose solution, and the amounts of clean water and sucrose solution expended were recorded. Sucrose preference can be calculated as follows:

7 $$\begin{aligned}&\mathrm{Sucrose}\;\mathrm{preference}\;\mathrm{percentage}\;(\%)\:=\:\mathrm{sucrose}\;\mathrm{solution}\;\\&\mathrm{consumption}\;(\mathrm g)/(\mathrm{sucrose}\;\mathrm{solution}\;\mathrm{consumption}\;\lbrack\mathrm g\rbrack\:\\&+\:\mathrm{water}\;\mathrm{consumption}\;\lbrack\mathrm g\rbrack)\:\times\:100\%.\end{aligned}$$

### Open field test

The rats freely explored the surroundings for 5. 5 min after being placed in the center of a 100 × 50 cm black square cage, with the first 30 s utilized to acclimatize to the environment. The camera footage was used to calculate the time spent in the middle of the open field box and the overall distance traveled throughout the 5.5 min test [52].

### Novelty-suppressed feeding (anxiety-based test) (ABT)

The intrinsic fear of novelty in the rat is exploited to induce inhibition of feeding behavior caused by exposure to a new factor with this test [53]. In this model, animals starved for 24 h were exposed to a clear cage with a sawdust-covered floor, a single food pellet, and concentrated illumination. This test detects depression and anxiety-related behaviors by evaluating the time rats take to display a feeding behavior in response to a novel stimulus, as well as the amount of food consumed [54].

## Pharmacokinetic studies

### Animals

A total of 15 Wister male rats of average weight between 180 and 200 g were used to perform a comparative PK analysis between rectal and oral formulations. The rats were assigned into three groups at random (5 rats each, n = 5). To avoid defecation throughout the experiment, the rats were deprived of any food for 16 h before the commencement of receiving the rectal drug formulations, with only water permitted [55].

### DXH administration to rats

Group I and Group II received the optimized DXH-GLYS thermosensitive in situ gel and free DXH in situ gel rectally, respectively, whereas Group III received oral DXH solution at a dose of 20 mg/kg [32]. The anus was glued after dosing using adhesive tape for preventing any possible leakage of the administered dose [10]. The drug was present at an 8 mg/ml concentration in any gel preparation.

### Sample collection

At the intermissions of 0.5, 1, 2, 4, 6, 12, 24, 48, and 72 h, approximately 1 ml blood samples were obtained from the rats’ retro-orbital plexus and taken into tubes that contained EDTA solution. The EDTA tubes were then put in a centrifuge at 3000 rpm for 30 min, followed by the separation of the plasma from the blood completely. Then, at -20 °C, the separated serum samples were frozen until they were analyzed for DXH content.

### LC–MS/MS analysis of DXH in plasma

A modified Liquid chromatography-tandem mass spectrometry LC–MS /MS (Shimadzu, Japan) method was used for DXH estimation in rat plasma samples using venlafaxine as an internal standard [32]. The LC system is fitted with a *DGU-20A3* degassing unit, and an *LC-20AD* solvent distribution unit with a *SIL-20A* auto-sampler. Separation of DXH and venlafaxine was performed on a C18 column, 100 A (50 × 4.6 mm) (Phenomenex, USA), with a particle size of 5 mm. The mass spectrometer was an AB Sciex API-3200 (Foster City, CA, USA), used for quantitation in positive ion mode. The ion spray voltage was set to be 5500 V. A mixture of 80% (v/v) of acetonitrile and 20% (v/v) of 0.1% aqueous formic acid was used as the mobile phase. The column delivered the mobile phase at a 0.2 ml/min rate through the column. Multiple Reaction Monitoring mode was employed for mass quantification of DXH and internal standard at m/z 298.01 to 153.95 for DXH and m/z 278.11 to 121 for venlaf axine.

### Sample preparation for analysis

The extraction of DXH from all thawed plasma samples was accomplished using a liquid–liquid extraction technique. 500 µl of each rat plasma was spiked with 50 µl of venlafaxine (100 ng/ml in acetonitrile), followed by the addition of 5 ml chloroform. To get the DXH in chloroform, the mixtures were vortex assorted and then subjected to centrifugation at 5000 rpm for 10 min. The upper layer had to be carefully removed, and the chloroform layer was taken into another fresh tube, then evaporated until completely dry. The dried test tube layer was then dissolved in 100 µl of methanol, and a 20 µl aliquot of this reconstituted clear extract was injected into the LC–MS/MS system for DXH quantification [56]. The same procedure was used to prepare all the plasma samples, either those used for calibration or PK analysis. A calibration curve for DXH in plasma has been constructed from 0.5 ng/ml to 250 ng/ml.

## Pharmacokinetic analysis

WinNonlin software (version 1.5, Scientific consulting, Inc., Rockville, MD) was used to estimate the various PK characteristics of DXH from the data obtained for each rat. From the rat plasma concentration–time profile, C_max_ (ng/ml) (the maximum observed DXH plasma concentration) and T_max_ (h) (the time taken to achieve C_max_)), mean residence time (MRT), elimination rate constant (K), and biological half-life (T_1/2_) were attained using a standard non-compartmental PK model. Based on the linear trapezoidal rule, AUC_0—72 h_ (the area under the plasma concentration–time curve) and AUC_0-∞_ were obtained. Finally, the relative bioavailability (F_rel_) for the rectal preparations was computed depending on the following formula:

8 $$\begin{aligned}\mathrm{Frel}=&\;\mathrm{AUC\ of\ in\ situ\ gel\ formulation/AUC\ of}\\&\mathrm{ oral\ formulation\ *100}\end{aligned}$$

## Histological study

On two groups of male albino rats (three rats per group, n = 6), a rectal histopathological evaluation was performed. The rats fasted for 24 h (with free access to water) before the commencement of the histological experiment to minimize fecal matter in the rectum. Group A was the control, whereas group B received the optimized DXH-GLYS in situ gel rectally. Rats were killed 24 h after drug administration. Then, the rectum segments were separated, fixed in 10% formalin buffer, and eventually entrenched in paraffin and sliced into sections. Finally, the paraffin rectum slices were stained with hematoxylin and eosin, and any abnormalities or irritation were visualized under an optical microscope [23].

## Statistical analysis

SPSS version 22 software (Chicago, IL) was used for statistical analysis of the obtained data. The significant difference between the PK parameters attained for both the rectal gel formulations and oral DXH solution was substantiated using a one-way analysis of variance (ANOVA) followed by the Tukey multiple comparisons test. Mean differences were statistically significant when the level of probability was *p* < 0.05. All the obtained data were represented as the average ± standard deviation of 3 experiments.

## Results and discussions

### Characterization and optimization of the DXH-GLYS formulations

Design of experiments is a combination of various techniques, successfully used to investigating the impact of variables as well as their interactions on the experimental outcome. Response surface methodology is a statistical and mathematical approach used widely in pharmaceutical research to detect a functional relationship among several responses and variables [57]. It could concurrently optimize the studied factor levels to produce a formulation having the required desirable properties and efficacy. Box–Behnken design was applied in the present study since it required significantly fewer experiments in comparison to other techniques such as a full-factorial design [58].

The coefficient of determination (*R*^2^), adjusted (*R*^2^), predicted (*R*^2^), and CV% values were employed to evaluate the best fit model for each response. Table S1 shows the quadratic values of the dependent variables alongside their R^2^, SD, and %CV.

### DXH entrapment efficiency

The nanoglycerosomal carrier successfully retained DXH, with an EE% fluctuating from 69.11 to 98.12%, as illustrated in Table 1. The quadratic model was deemed to be the best model that fits the data of EE% depending on its highest value of R^2^. It was statistically significant (*P* < 0.0001), with an F-value detected to be 151.64. According to the analysis of the experimental data by ANOVA, the following polynomial equation was generated:

9 $$\begin{aligned}\mathrm{EE}\%=&+86.99+7.02\mathrm A-8.20\mathrm B+2.47\mathrm C-1.30\mathrm{AB}\\&-1.73\mathrm{AC}-0.59\mathrm{BC}-1.06\mathrm A^2-3.62\mathrm B^2+0.82\mathrm C^2\end{aligned}$$

A positive sign for a regression coefficient represents a synergistic impact of the factors, whereas a negative sign for a regression coefficient represents an antagonistic impact. Both PL90G and Tween 80 concentrations displayed a synergistic effect (*p* < 0.0001) on the EE % of DXH. Notably, increasing PL90G concentration from 2 to 4% at constant glycerol and Tween concentrations increased the EE % of F5 from 81.79 ± 0.99% to 98.12 ± 0.73% in F11. Furthermore, the EE % of F2 was 75.38 ± 0.82%, which increased to 83.76 ± 0. 32% in F4 after increasing the concentration of Tween 80 from 5 to 15% while keeping PL90G and glycerol constant. The amplified EE% of DXH within GLYS by increasing PL90G could be credited to the high surface area available for trapping DXH at higher PL90G concentrations acquired from the lipid bilayer architecture of the closed vesicles of GLYS [59]. Also, the lipophilic nature of DXH, which makes it freely solubilized in PL90G, is mainly responsible for increasing the possibility of its integrating and depositing with the lipidic content of GLYS, therefore enhancing DXH EE% [38, 39]. Moreover, the larger glycerosomal size attained with increasing PL90G concentration might have resulted in a higher encapsulation volume for DXH [34, 60]. This finding coincides with that proposed by Zhang et al. (2017), who endorsed that increasing phospholipid concentration increased paeoniflorin EE% in glycerosomal vesicles [59]. Similarly, Tween 80 had a synergistic effect on solubilizing poorly soluble drugs like DXH that increased the drugs' wettability, subsequently increasing the lipophilic bilayer region volume available for DXH entrapment [61, 62].

**Fig. 1:**
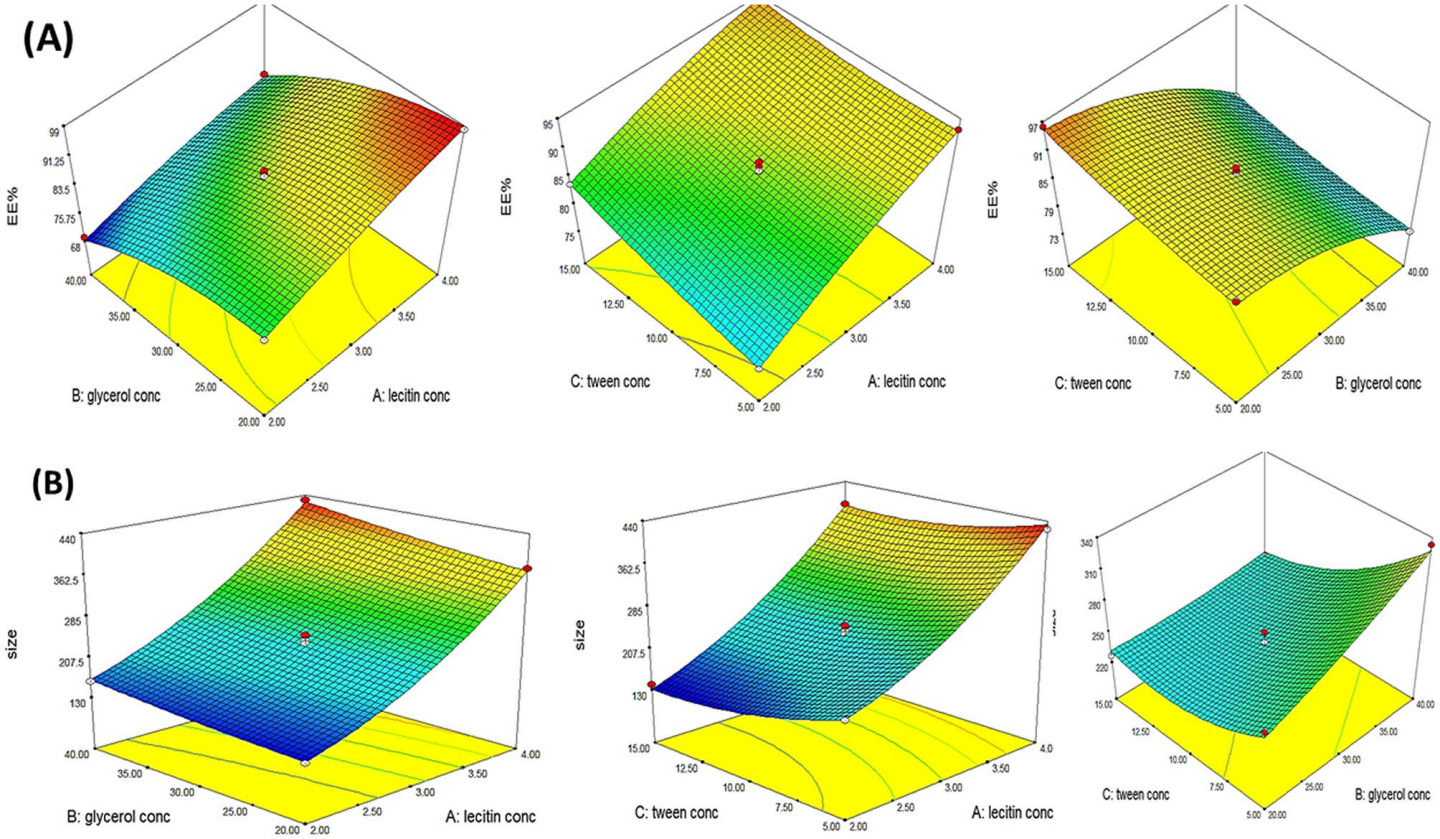
(**A**) 3D-response surface plot showing effect of independent variables on (**A**) % entrapment efficiency (EE%) and (**B**) particle size (PS)

Furthermore, the EE% of DXH exhibited a significant negative correlation (p < 0.0001) with glycerol percent (C), where the EE% of F1 that had 40% glycerol and F11 that had 20% glycerol were 80.22 ± 0.95% and 98.12 ± 0.73%, respectively. The increase in the DXH solubility in the intervesicle medium containing glycerol upon increasing the glycerol content in the water phase could explain the seepage of DXH from GLYS and the decrease in % EE at higher glycerol percentages [27, 63]. Manca et al. (2014) shared similar findings, as they prepared quercetin GLYS to enhance drug protective action against oxidative stress. The impact of PL90G, Tween 80 concentrations, and glycerol percentage on EE% was represented by a 3-D plot, Fig. 1A.

### Vesicle size analysis

Vesicles’ permeation through the rectal mucosa was significantly affected by their particle size, and, therefore, the preparation of small-sized GLYS could greatly improve drug absorption. The vesicle size of DXH-GLYS formulations varied dramatically from 135.9 ± 2.32 to 430.6 ± 3.9 nm, with acceptable PDI values (PDI < 0.7), indicating good uniformity. The impact of the independent variables on the vesicle size of GLYS was fitted to the quadratic model depending on its highest value of R^2^. The model F-value of glycerosomal size was 171.68 (significant at ***p*** = 0.0001). Equation 9 represented the quantitative influence of the three factors on the size of GLYS with the coded values.

10 $$\begin{aligned}\mathrm{GLYS\;size}=&+241.97+123.71\;\mathrm A+20.55\;\mathrm B-27.44\;\mathrm C\\&+7.48\;\mathrm{AB}+7.70\;\mathrm{AC}-15.82\;\mathrm{BC}+29.94\mathrm A^2\\&+3.87\mathrm B^2+18.24\mathrm C^2\end{aligned}$$

The positive sign of A and B indicates that PL90G concentration and glycerol percent evinced a significantly synergistic impact on the size of DXH-GLYS (*p* < 0.0001). The vesicle sizes of F1 comprising 4% PL90G and F3 comprising 2% PL90G were 430.6 ± 3.9 nm and 160.9 ± 2.97 nm, respectively. Also, the vesicle sizes of F1 having 40% glycerol and F11 having 20% glycerol were 430.6 ± 3.9 nm and 375.7 ± 2.0 nm, respectively. The increased GLYS size with increasing PL90G concentration might be attributed to the tendency of PL90G molecules for aggregation and coalescence by increasing their concentration, occasioning the formation of large-sized GLYS [64]. Another hypothetical interpretation for the enlargement in the GLYS size is that the viscosity of GLYS was increased with increasing PL90G concentrations, which decreased the homogenization effect on vesicles and the ultrasonic power efficacy for vesicle size reduction [65]. Such finding was consistent with previous researches [22, 66, 67].

Similar to the PL90G effect, the increase in glycerol percentage from 20 to 40% boosted the size of GLYS significantly. This could be owing to the glycerol's sticky nature [30, 59]. On the contrary, the Tween 80 concentration (C) demonstrated a significant negative effect on the glycerosomal size (*p* = 0.0064), where the vesicle sizes of F2 and F4 were 200.2 ± 5.67 nm and 140 ± 1.25 nm, respectively. A possible explanation for this observation is the ability of Tween 80 to reduce the interfacial tension between the lipid and aqueous components, resulting in a decline in the vesicular size [68]. The influence of PL90G, Tween 80 concentrations, and glycerol percent on vesicle size was represented by a 3-D surface plot as shown in Fig. 1B.

## In vitro release studies

According to the ANOVA test, the quadratic interaction model was significant (***p*** = 0.0006) for the observed % CDXR data, with an F value of 34.88, which fitted the data well. The obtained data revealed that the different prepared GLYS formulations efficiently released DXH in a sustained pattern (from 30.96 ± 2.5% to 60.52 ± 1.51% over 24 h), whereas the DXH solution rapidly released 100% of the native drug in 4 h. This finding was in line with previous studies on DXH [6]. In an earlier reported study, DXH released from the formulated proniosomal gel and mucoadhesive proniosomal gel after 24 h was informed to be 30% and 24%, respectively. This demonstrated that our DXH-GLYS formulations achieved higher DXH levels and better-sustained release patterns compared to earlier studies. The quadratic model of the % CDXR data was represented in terms of the coded factors by the following polynomial equation:

11 $$\begin{aligned}\%\mathrm{CDXR}=&+51.69-8.51\;\mathrm A-6.55\;\mathrm B+1.67\;\mathrm C+3.87\;\mathrm{AB}\\&+2.53\;\mathrm{AC}-0.14\;\mathrm{BC}-6.61\mathrm A^2-1.65\;\mathrm B^2+0.35\mathrm C^2\end{aligned}$$

**Fig. 2:**
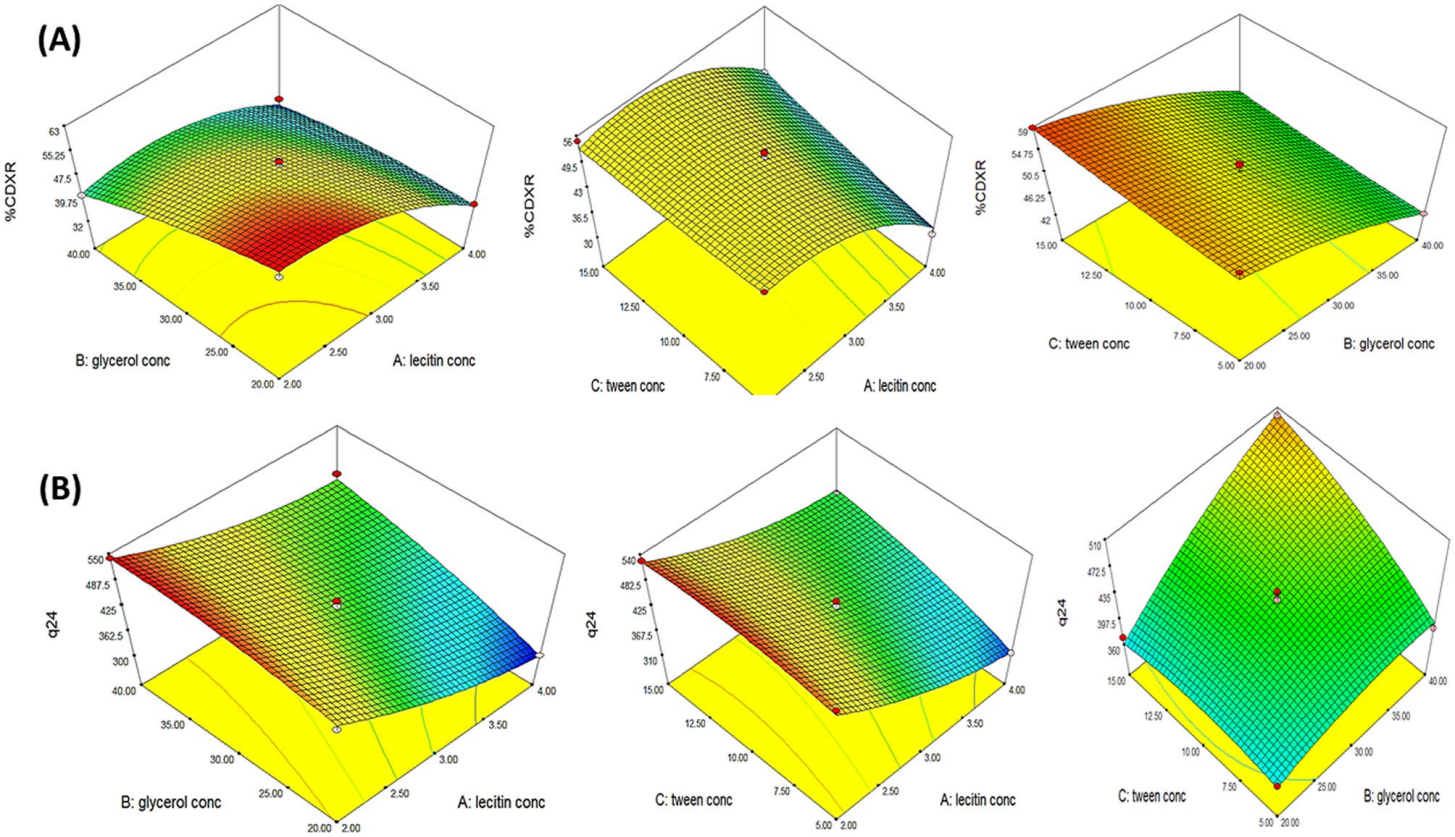
3D-response surface plot showing effect of independent variables on (**A**) %CDXR and (**B**) Q_24_(μg/cm^2^)

The negative sign of term A revealed that the PL90G concentration had a significant antagonistic effect on the %CDXR (p < 0.0001). The %CDXR of F1 comprising 4% PL90G and F3 comprising 2% PL90G were 34.08 ± 2.00% and 41.17 ± 1.42%, respectively. The decline in the %CDXR with the growing PL90G concentration is not surprising. This depressing impact might be imputed to forming long cylindrical micelles at higher concentrations of PL90G, forming a network structure that retained the drug. Consequently, the amount of DXH available to be diffused was reduced [69]. Likewise, by observing the impact of glycerol percent on % the CDXR, it was disclosed that the glycerol percent exhibited a significant negative effect on the % CDXR (*p* < 0.0001), where the % CDXR of F10 having 20% glycerol and F14 having 40% glycerol were 58.83 ± 0.89% and 43.96 ± 1.20%, respectively. This outcome could be succumbed to the osmotic property as well as the hygroscopicity of glycerol, which enables it to attract water from the releasing medium to the donor compartment, causing the concentration of water/glycerol mixture to become unbalanced, resulting in additional drug entrapment in GLYS and lessened DXH efflux. Furthermore, as previously outlined in the EE% evaluation, the increase in glycerol percentage ensued in a large vesicle size, as a result, reduced the surface area available for DXH release [27, 28]. Indeed, the drug release from GLYS was highly influenced by their particle size. The release rate of small-sized vesicles was higher than that of larger ones [70]. Tween concentration (C) evinced a moderate influence on % CDXR (p = 0.0607). The outcome of PL90G, Tween 80 concentrations, and glycerol percent on % CDXR was graphically depicted by the 3-dimensional surface plot in Fig. 2A.

Linear regression analysis was used to analyze the DXH release data to determine the release order of DXH-GLYS. Depending on the coefficient of determination with the highest value (R^2^), the release of DXH from different GLYS formulations has complied with Higuchi’s diffusion-controlled mechanism, which is the general release model for almost all nanovesicular carriers.

### Ex vivo skin permeation studies

*Ex-vivo *permeation analysis is a beneficial approach for predicting and giving some indications about the in vivo efficacy of drug delivery systems [71]. The rectum epithelium is commonly known to be similar to that of the upper GIT, and transcellular permeation appears to be the main mechanism of drug penetration across the rectal mucosa [72]. The DXH permeation parameters of DXH-GLYS were computed and manifested in Table S2. Figure S1 illustrated that the DXH-GLYS formulations have significantly higher DXH permeation across the rectal mucosa per unit area after 24 h (Q_24_ ranged from 300.23 ± 7.00 to 540.07 ± 19.57 μg/cm^2^) than the DXH solution with the same DXH amount (Q_24_ 223.03 ± 4.7 μg/cm^2^). The influence of the independent variables on Q_24_ was fitted to the quadratic model depending on its greatest value of R^2^. The quadratic model that was proposed had an F-value of 52.13 and was statistically significant (*p* = 0.0002). Equation (10) denotes the outcome of independent variables on Q_24_ in coded values:

12 $$\begin{aligned}{\mathrm Q}_{24}=&+432.37-71.50\;\mathrm A+41.17\;\mathrm B+24.77\mathrm C+17.87\;\mathrm{AB}\\&+16.99\;\mathrm{AC}+27.39\;\mathrm{BC}+28.57\mathrm A^2-12.93\mathrm B^2-14.02\mathrm C^2\end{aligned}$$

With respect to Eq. 10, the observed negative sign of term (A) revealed that increasing PL90G concentration had a significantly negative impact on Q_24_ of DXH (*p* < 0.05), where Q_24_ of F2 having 2% PL90G and F9 having 4% PL90G were 522.21 ± 18.03 and 332.5 ± 10.99 µg/cm^2^, respectively. Generally, the presence of PL90G, which has a strong affinity for biological membranes, as the main component of GLYS helps enhance DXH permeation efficiency. A notable finding was that the Q_24_ values of DXH-GLYS were found to decrease simultaneously as the PL90G content was increased. This discrepancy might be argued on the basis of formation of less deformable and more intact large-sized GLYS formed at higher PL90G concentrations. Therefore, due to the resultant rigidity of the developed GLYS at higher PL90G levels, DXH was not able to cross the rectal mucosa [73, 74]. These results were in line with those gathered from the in vitro release study. In a parallel line, the escalation in DXH EE% resulting from the increased PL90G concentration could indirectly result in a decrement in the vesicles’ flexibility, which is interrelated to the restriction in phospholipid fluidity caused by the drug molecules, thereby reducing DXH permeation [75, 76].

A significant synergistic effect was observed for Tween 80 concentration and glycerol percentage on Q_24_ of DXH as evidenced by the positive signs of (B) and (C) in the above equation. Q_24_ of F9 having 5% Tween 80 and F13 having 15% tween 80 were 315.5 ± 10.99, 405.6 ± 9.85 µg/cm^2^, respectively, whilst Q_24_ of F10 containing 20% glycerol and F14 containing 40% glycerol were 370.45 ± 7.0, 500.32 ± 15.29 µg/cm^2^, respectively. The significantly boosted permeability of DXH across the rectal mucosa after 24 h from the assembled GLYS by increasing Tween 80 and glycerol concentration (edge activators) can be correlated to enhanced flexibility of the developed GLYS by increasing their concenterations, which enhanced the DXH release from the nanovesicles and thus facilitated DXH diffusion through the rectal epithelial [77, 78]. The deformability and fluidity of the phospholipid bilayer of GLYS also enabled them to squeeze through the rectum biological membrane more easily [35, 79]. These novel GLYS appear to combine the features of both elastic vesicles and penetration-enhancer contained vesicles [80–82]. The influence of PL90G, Tween 80 concentrations, and glycerol percent on Q_24_ (µg/cm^2^) was graphically depicted by a 3-D surface plot in Fig. 2B.

## Optimization

Regarding data analyzed by ANOVA, there was no significant lack of fit for any response. The desirability approach and numerical optimization were employed for determining the optimum GLYS formulation composition. Design Expert^®^ software has suggested A, B, and C levels based on maximizing Y1, Y3, Y4, and minimizing Y2 to prepare the optimized formulation according to the following composition: 2% w/v PL90G, 23.01% v/v glycerol, and 15% w/w Tween 80. This composition was selected depending on the formulation, with a high desirability index (0.828){Venugopal, 2016 #37;Venugopal, 2016 #37}. The noticed responses attained from the new prepared formulation were in close agreement with the predicted values with an acceptable percentage error, as evinced in Table 2. Moreover**,** the observed and predicted values were not significantly different at ***P*** ≤ 0.05, indicating that the optimization process was reliable. The PDI value of the optimized GLYS formulation was 0.348 ± 0.09, demonstrating good dispersion homogeneity.

**Table 2:** Predicted and experimental values of the optimized DXH nano-glycerosomal formulation (means ± SD, *n* = 3)

	phospholipon 90G concentration (%w/v)	Glycerol percent	Tween concentration (%w/w from the total lipids)	Y_1_(%)	Y_2_(nm)	Y_3_(%)	Y_4_(µg/cm^2^)	Desirability
		(%v/v)						
Predicted value	2%	23.01%	15%	87.4	135.14	59.67	484.15	0.822
Experimental value	2%	23.01%	15%	86.66 ± 1.48	130.34 ± 16.22	58.44 ± 1.08	475.34 ± 7.08	0.822
Bias%	––	––	––	0.85	3.68	2.1	1.85	––

%Bias = (predicted value-experimental value)/experimental value

### In vitro release evaluation of the optimized DXH-GLYS formulation

**Fig. 3:**
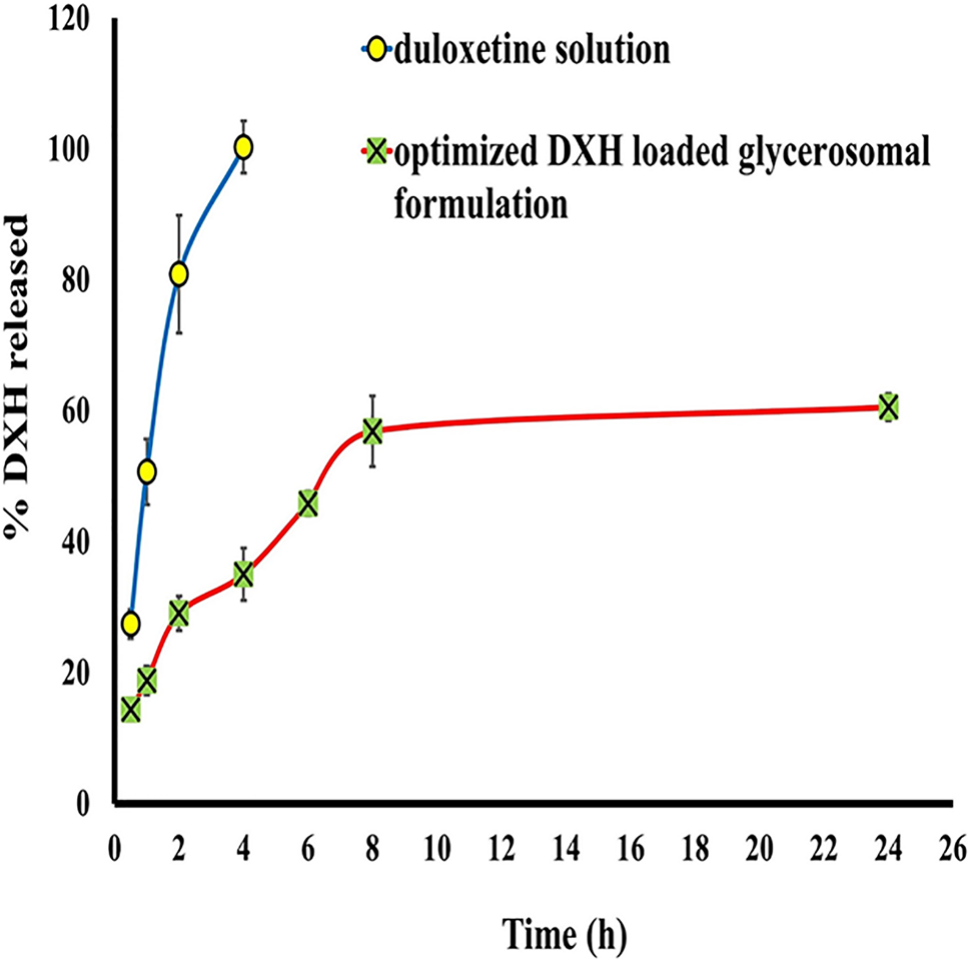
% Cumulative DXH released from the optimized nano-glycerosomal formulation against oral DXH solution in phosphate buffer pH.7.4

Figure 3 demonstrated that the optimized DXH-GLYS formulation had a sustained release behaviour for DXH compared to the DXH solution.

### Morphology of the optimized DXH-GLYS formulation

**Fig. 4:**
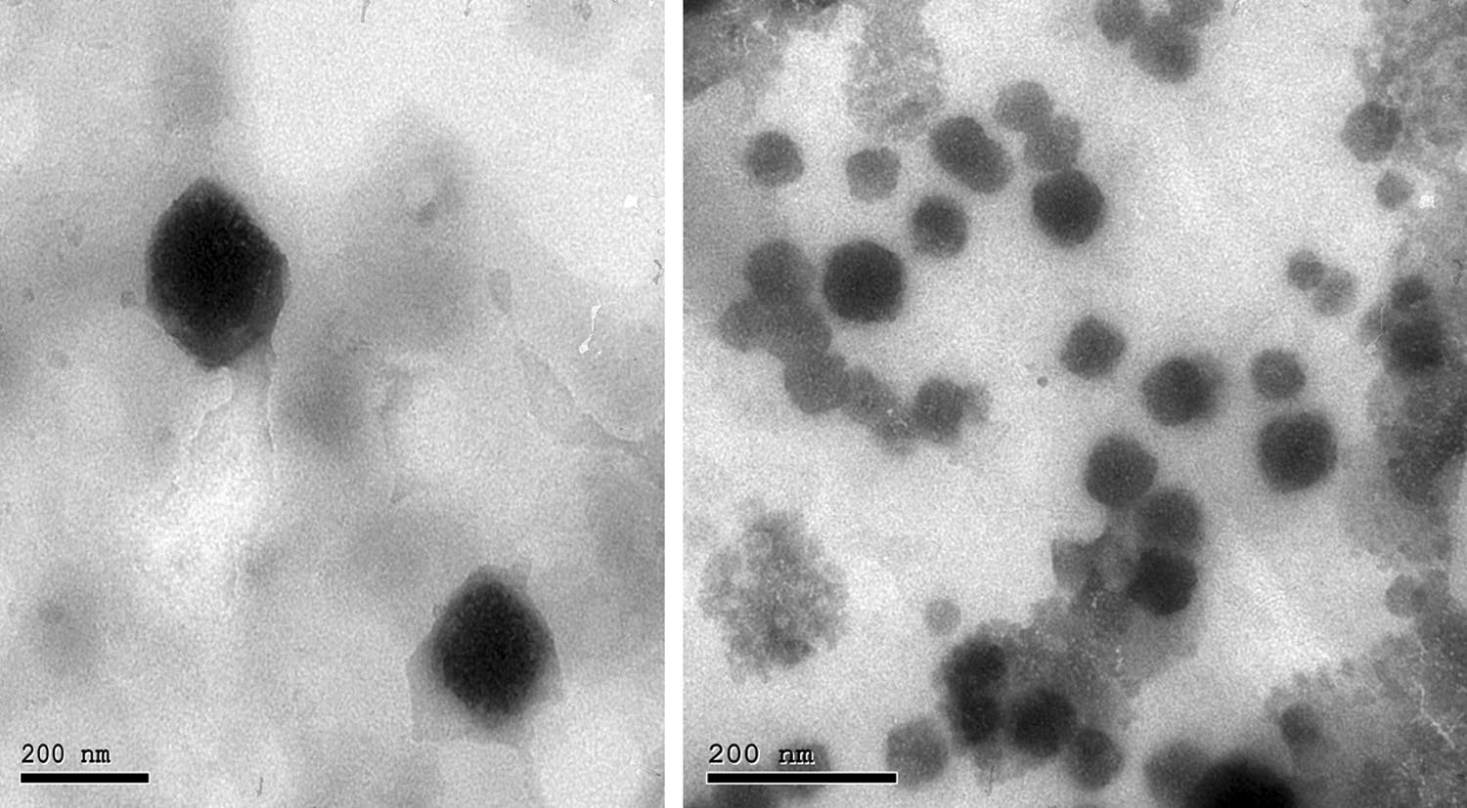
TEM photomicrograph of the optimized DXH nano-glycerosomal formulation

The optimized DXH-GLYS formulation appeared by TEM as black dots, representing vesicles with sphere-shaped and smooth surfaces, without any agglomerations or DXH crystals, as depicted in Fig. 4. TEM images revealed vesicles with a nanometric size, closely related to the vesicle size detected utilizing the DLS approach.

### ZP of the optimized DXH-GLYS formulation

ZP is used as an indicator for the stability of GLYS, reflecting the proclivity of nanoparticles for agglomeration. The optimized formulation has a high ZP (+ 15 mV), indicating vesicle stability. As previously reported in the literature, DXH has a cationic moiety, so adsorption of the drug on the surface shifts the ZP of GLYS vesicles toward the positive side [6, 32].

### Physical stability of the optimized DXH-GLYS formulation

After 90 days of storage, it can be detected from the stability study that the optimized DXH-GLYS formulation did not differ significantly from the freshly prepared one in terms of EE%, glycerosomal size, or ZP (*p* < 0.05). No precipitation or layer separation was noticed in the optimized formulation, indicating that it was physically stable.

### Evaluation of the DXH-GLYS in situ gel

The fabricated DXH-GLYS in situ gel was elegant, white, homogenous, and smooth with no air bubbles or lumps. The rectal mucosa could endure preparations having a pH of 6.8–7.4 [9]; meanwhile, the pH value of the optimized DXH-GLYS in situ gel was found to be 7.05, and thus, it was rectally tolerable and expected to be free of any pH-related harmful impacts. Further, the DXH content percentage of the in situ gel was 98.92 ± 1.19%, indicating good homogeneity. The spreadability of the in situ gel formulation also plays a crucial role in the gel’s efficacy as a drug delivery system that could be accredited to increasing the surface area available for DXH absorption at the administration site. The gel formulation was easily spreadable (diameter 5.7 cm). Moreover, the assembled DXH-GLYS in situ gel had a gel strength of 45.7 s, demonstrating that the in situ gel can be considered reasonable for rectal use.

Indeed, the ideal in situ gelation temperature range was from 30 to 36 °C. The DXH-GLYS in situ-gel was transformed from a liquid state to a gel state after 30 s when maintained at 33 ± 0.09 °C, and remained intact for a few hours (+ +) according to the coding for the gelling capacity that was previously mentioned. Such a short gelation time of the formulation suggests that it could form a gel quickly after rectal administration, preventing the leakage of the dosage form from the anus. Additionally, the bioadhesive force of GLYS in situ gel was calculated to be 14,582.8 ± 2.39 dyne/cm^2^, which was high enough to prevent the gelled formulation from reaching the colon's end, where the first-pass effect occurs. These results could be correlated to the high molecular weight and the viscosity of the entrenched HPMC, since higher molecular weight is critical in maximizing bioadhesion via entanglements and van der Waals attractions [43].

In addition, the optimized DXH-GLYS in situ gel had a viscosity value of 1113.28 ± 10.65 at 25 °C and 6679.7 ± 9.22 cps at 37 °C with Farrows constant greater than one (*N* = 3.43), confirming shear thinning characteristics of the developed nanoglycerosomal gel, Figure S2. The area of the hysteresis loop was also calculated to be 2449.216 (Dyne/cm^2^.sec). Presumably, a higher shear rate provokes lower viscosity and vice versa. Since the rectum is constant and static compared to the upper GIT, the shear rate is believed to be minimal, and the formulation's maximum viscosity will remain unchanged after administration, which will aid in avoiding gel leaking from the administration site [8].

Regarding the in vivo localization test, the blue color of the optimized DXH-GLYS in situ gel formulation was clearly detected in the rectum after 5 min from the administration. Then, after 6 h, the blue color of the in situ gel was also noticed in the rectum 4 cm above the anus, but faded. This indicated that the gel’s position in the rectum did not change significantly over time and was not observed in the colon. These outcomes suggested that the mucoadhesive force of the gel formulation was sufficient to keep them in the rectum for more than 6 h.

**Fig. 5:**
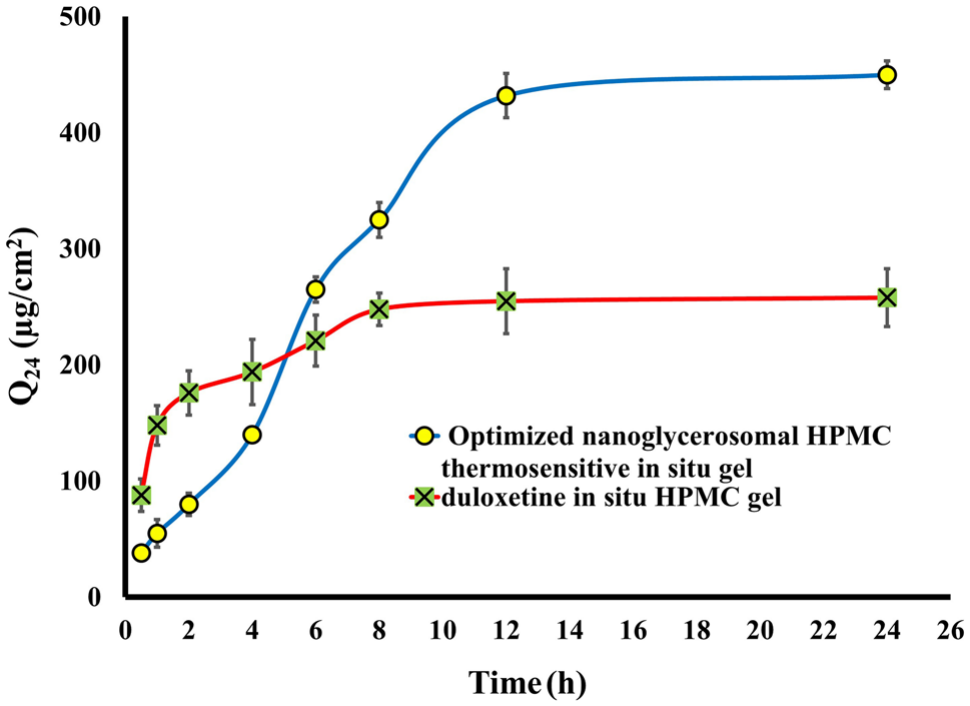
Cumulative DXH permeated per unit area after 24 h (Q_24_ μg/cm^2^) of optimized DXH nano-glycerosomal loaded thermosensitive in situ gel compared to free DXH loaded in situ gel

Concerning the in vitro release and ex vivo permeation studies, the obtained results disclosed that the dispersion of both free drug and drug-loaded GLYS in the in situ poloxamer gel resulted in a decrease in the released and permeated amount of DXH from both. This attitude can be ascribed first to the “dual reservoir effect," which occurs when the vesicles are incorporated into a gel matrix as a drug reservoir, preventing drug diffusion from the gel system to the surrounding media [83]. Second, the formulation’s viscosity also affected the amount of DXH released or permeated because the drug was released from the gel matrix via diffusion across the extramicellar aqueous channels, and the gel’s high viscosity hinders this process [84]. On the other hand, the permeation parameters of the DXH-GLYS in situ gel formulation (Q_24_ = 450.25 ± 12.54 µg/cm^2^ permeated after 24 h, Jss = 30.42 μg/cm^2^/h) were significantly higher than those of the free drug gel (Q_24_ = 199.55 ± 20.11 µg/cm^2^, Jss = 11.586 μg/cm^2^/h), which contribute to the permeation-enhancing effect of the incorporated GLYS as described previously, Fig. 5. The enhancement ratio was 2.62. To our knowledge, a previous report informed that the flux of DXH from intranasal DXH mucoadhesive proniosomal gel was found to be 16.1 μg/cm^2^/h [85]. According to our findings, the DXH-GLYS in situ gel showed a flux of 30.42 μg/cm^2^/h. This result revealed our formulation's superiority over earlier studies in increasing DXH permeation across the rectal mucosa.

## Pharmacodynamics studies

Serotonin and norepinephrine insufficiency is a clinical sign of depression, characterized by decreased functioning and well-being, slowness of movement, and excessive work absenteeism [86]. In rats, researchers have assessed mobility to distinguish between drug effects, define the functional roles of various neurobiological systems, and screen for compounds with potential psychoactivity [56, 87].

Following treatment with DXH-GLYS, we observe a significant increase in behavioral activity attributable to the neurotransmitters restoration. DXH raises serotonin and norepinephrine levels in the rat frontal cortex and hypothalamus by inhibiting their reuptake into the presynaptic neuron, thereby retaining the molecules in the synaptic gap longer. Since the neurotransmitters remain in the synapse for an extended time, they continue to bind to the postsynaptic neuron’s receptors, provoking the physiological effects associated with them. The enhancement of neurotransmitters exclusively increases climbing and swimming time and various behavioral activities in rats.

### The forced swimming test (FST)

**Fig. 6:**
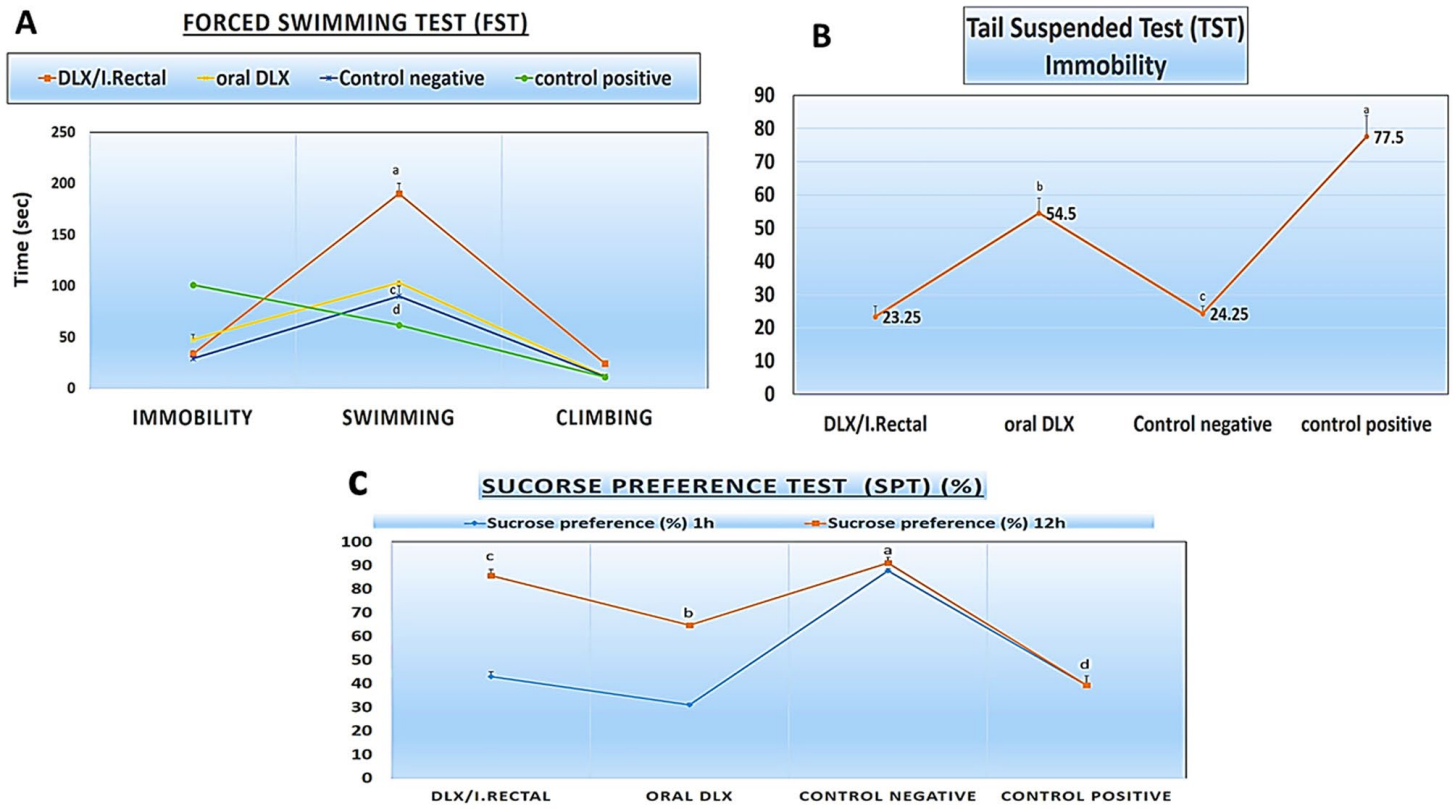
**A**. Behavioral analysis (Immobility, Swimming & Climbing) after forced swimming test (FST) for two preparations (oral & rectal) in comparison to normal and depressed rats. **B**. Tail suspended test (TST), immobility for two preparations (oral & rectal) in comparison to normal and depressed rats. **C**. Effect of food deprivation as a mild stress on the sucrose preference percentage in rats to the two preparations (oral & rectal). Sucrose preference measured within 1 h (blue) and within 12 h (red). Depressed rats showed decreased in the sucrose preference in rats. Results are presented as the mean ± SD, *P* < *.05*, compared with the control negative (normal rats) and control positive (depressed rats) group

FST is a behavioral test extensively used in rats to assess the potential efficacy of new antidepressant medications and predict the success of antidepressant therapy in humans [88]. The swimming test works on the idea of generating immobility, a depression-like behavior, and then determining if antidepressant medications can reverse it. As depicted in Fig. 6 A, there was a significant reduction in immobility time by treating the animals with DXH. The mean value of immobility time for the two treated groups (oral and rectal) was found to be significant when compared to normal or depressed groups (*p* < *0.05*). Rectally administered rats demonstrated a significant decrease in the total immobility period (33.5 ± 3.1 s) in comparison to the oral solution treatment group (69 ± 4.5 s). Also, after 24 h of investigation, the rats in the DXH rectal gel group climbed (24 ± 1.1 s) (*p* < *0.05*) and swam (190 ± 10 s) (*p* < *0.05*) more than the rats in the DXH oral solution and control groups.

### Tail suspension test

The tail suspension test revealed that rats treated with the DXH-GLYS in situ gel had a significantly lower immobility time (23.25 ± 3.3 s) than rats treated with the oral DXH solution (54.5 ± 4.4 s) *(p* < *0.01*), indicating that DXH rectal dosing has better antidepressant activity than DXH oral dosing, Fig. 6B. That could be related to the maintenance of constant plasma DXH concentration by rectally administering DXH-GLYS.

### Sucrose preference test (SPT)

The sucrose preference percentages of rats assessed after 1 h and 12 h were compared, and the results are presented in Fig. 6C. The percent value after 12 h was greater than that after 1 h. The rectal administration had the highest sucrose preference (85.66% ± 2.08) when compared to the oral DXH solution group (64.6% ± 0.5) and its effects were very similar to those of the negative control group.

### Open field test (OFT)

The depressed control rats had considerably *(p* < *0.05*) lower overall distance traveled and percentage of central zone distance (1503 ± 3.55 cm & 0.9 ± 0.1%) than the normal control rats (2063 ± 1.13 cm & 3.6 ± 0.5%). Meanwhile, the rectal DXH-GLYS in situ gel treatment group had a substantially higher overall distance traveled and % central zone distance (2583 ± 5.0 cm & 7 ± 0.9%) than the oral DXH solution (2283 ± 2.8 cm & 4.6 ± 1.5%) and normal control groups (2063 ± 1.13 cm & 3.6 ± 0.5%), Figure S3.

### Novelty suppressed feeding (anxiety-based test) (ABT)

Antidepressant-treated rats, particularly those administered via the rectal route, demonstrated a considerably shortened latency to feed in a new situation (latency to eat in the novel environment) (60.3 ± 4.5 s) than the negative control (63.6 ± 3.2 s) and positive depressed groups (82.3 ± 2.5 s), as well as when compared to the oral DXH solution group (73.6 ± 3.2 s), Fig. S4.

## Pharmacokinetics studies

**Fig. 7:**
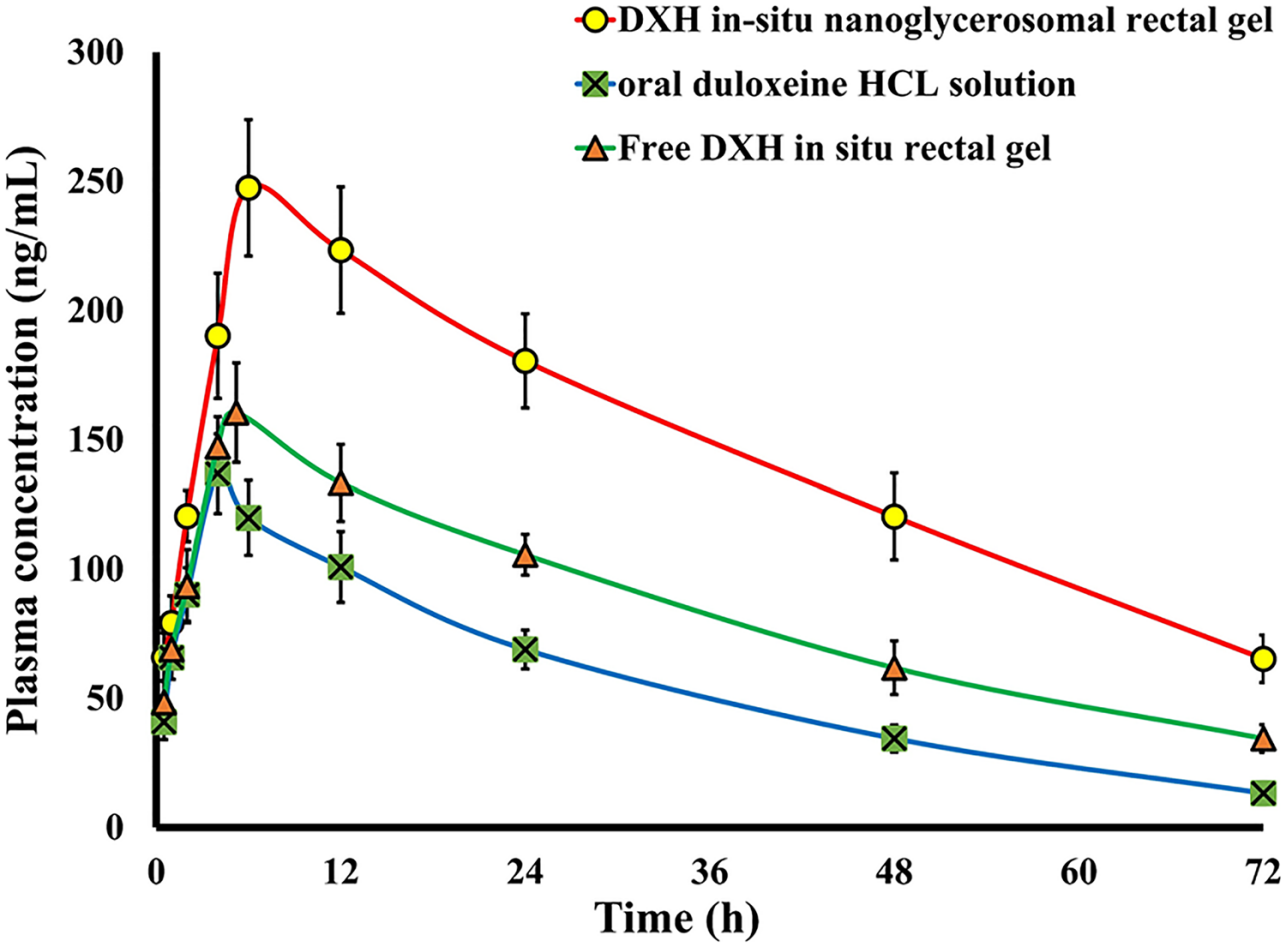
Plasma concentration–time profiles of duloxetine HCl in rats after administration of oral DXH solution, rectal optimized DXH nano-glycerosomal in situ gel and rectal free DXH loaded in situ gel (mean ± SD)

To study the in vivo performance of the optimized DXH-GLYS in situ gel, the free DXH in situ gel, and make comparisons with oral DXH solution, the LC–MS/MS assay was utilized to quantify DXH in order to estimate the pharmacokinetic parameters of DXH in rat plasma. Figure 7 depicts the mean plasma DXH concentration–time profile following oral DXH solution administration and rectal application of the optimized DXH-GLYS in situ gel and free DXH in situ gel. The resulting pharmacokinetics parameters are outlined in Table 3. As obvious, C_max_ value of the rectal DXH-GLYS in situ gel (247.75 ± 26.37 ng/ml at T_max_ = 6 h) was significantly higher (*p* < 0.0001) than that of the oral DXH solution (136.86 ± 19.42 ng/ml at T_max_ = 4 h). Furthermore, maximum concentration of 160.46 ng/ml was reached at 5.2 h for rectal free DXH in situ gel. The plasma level of the oral DXH solution was dropped quickly in the hours following administration. On the other hand, the rectal formulations sustained the DXH plasma concentration over time. The calculated AUC_(0–∞)_ of the rectal GLYS in situ gel (13,699.63 ng.h/ml) and free DXH in situ gel (7962.48 ng.h/ml) was strikingly higher (*p* < 0.0001) (2.24-folds and 1.30 folds, respectively) when compared to that of the oral DXH solution (6106.6 ng.h/ml). The t_1/2_ was 18.36 ± 3.91 h for oral DXH solution, whereas it was 20.75 ± 4.6 h for rectal free DXH in situ gel and 25.91 ± 6.15 h for rectal GLYS formulation.

**Table 3:** Pharmacokinetics parameters of DXH loaded nanoglycerosomal in situ gel and oral DXH solution

**Pharmacokinetics parameter**	**Oral DXH solution**	**Rectal Free DXH gel**	**Rectal DXH Nanoglycerosomal in situ gel**
**C**_**max**_** (ng/ml)**	136.86 ± 19.42	160.45 ± 19.22	247.75 ± 26.37
**T**_**max**_**(h)**	4 ± 0.00	5.2 ± 0.97	6 ± 0.00
**T**_**1/2**_**(h)**	18.36 ± 3.91	20.75 ± 4.67	25.91 ± 6.15
**K**_**e**_**(h**^**−1**^**)**	0.037 ± 0.002	0.033 ± 0.003	0.026 ± 0.004
**MRT(h)**	30.47 ± 5.24	41.53 ± 6.4	8.29 ± 54.81
**AUC**_**( 0–72)**_** (ng.h/ml)**	5005.80 ± 1080.17	6112.00 ± 535.86	796.11 ± 10,736.11
**AUC **_**(0-∞)**_** (ng.h/ml)**	6106.67 ±785.2	7962.48 ± 797.76	13,699.63 ± 2076.8
**Relative bioavailability**		130.38	224.1

C_max_: Maximum serum concentration, T_max_: Time to reach C_max_, AUC_(0–72):_ Area under the serum concentration–time curve, and *MRT* Mean residence time

The delayed T_max_ and prolonged T_1/2_ for both rectal formulations relative to oral DXH solution inferred retardation in the DXH release which could be accredited to the following reasons: (i) the bioadhesive HPMC gel matrix had the aptitude of enhancing the overall product viscosity and distorting or squeezing the extra-micellar aqueous channels of poloxamer micelles through which DXH can diffuse [89]; (ii) the capability of HPMC in lowering the sol–gel conversion rate of both gel formulations as it binds to polyethylene oxide chains present in the poloxamer molecules, promoting rapid dehydration and enhancing entanglement of adjacent molecules with intermolecular hydrogen bonding; and (iii) the mucosal lining of the rectum is made up of oligosaccharide chains with sialic acid, thus, mucoadhesive polymers with hydrophilic groups like hydroxyl groups could bind powerfully to the oligosaccharide chains, producing a greater bioadhesive force with the rectum, and hence a delay in DXH release [13]. Therefore, the rectal administration of either GLYS in situ gel or free drug in situ gel showed a slow absorption profile for DXH, with delayed T_max_ until complete drug diffusion from the gel matrix to the surrounding environment and then to the systemic blood. In contrast, the significantly higher residence time of DXH on rectal mucosa exerted by the GLYS in situ gel as compared to free DXH in situ gel might be owed to the dual sustained effect induced by both the vesicular and gelling system [14]. These findings were consistent with DXH's in vitro release studies.

The relative bioavailability of DXH as a rectal GLYS in situ gel and free DXH in situ gel was 130.38% and 224.1% respectively, with respect to oral DXH solution. This remarkable increment in DXH bioavailability can be elucidated in terms of bypassing hepatic first-pass metabolism as well as surmounting DXH acidic hydrolysis in the GIT, both of which are accomplished via rectal route administration. Taken together, the mucoadhesive effect of HPMC and the base (Poloxamers) could have prevented the gelled formulation from traveling to the upper hemorrhoidal vein, which feeds into the mesenteric veins via the hepatic portal veins into the liver, improving lower rectum retention. Absorption through the lower hemorrhoidal vein, through which the drug feeds directly into the systemic circulation, could boost DXH bioavailability [90]. Furthermore, the penetration-enhancing effect of GLYS as a nanocargo was a significant factor in the increased DXH bioavailability as compared to free DXH in situ gel. The obtained results implied that the DXH-GLYS in situ gel could effectively deliver DXH rectally.

## Histopathological investigation

**Fig. 8:**
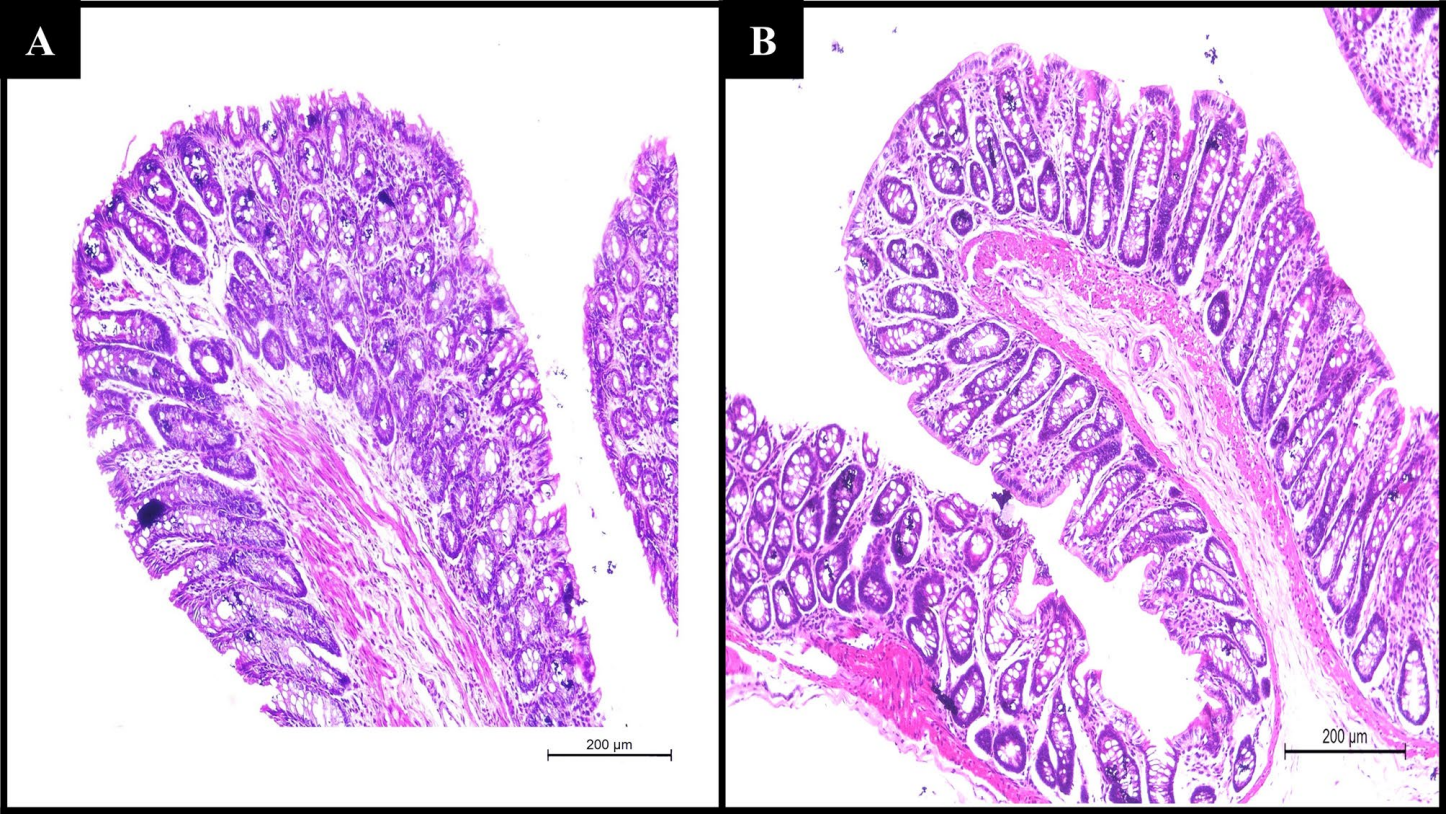
Histopathology photomicrographs of (**A**) untreated rectal mucosa and (**B**) rectal mucosa treated with optimized DXH-nanoglycerosomal in situ gel formulation

Histopathological examination was carried out on the rats’ rectum specimens to detect any irritation or damage observed in the rectal tissues after applying GLYS gel relative to the control, Fig. 8. Compared to the control group, normal rectal epithelium and intact covering epithelium with multiple goblet cells have appeared in the rectal mucosa of the group B receiving the GLYS in situ gel without any indications of adverse reactions (erythema, irritation, or inflammation) following rectal administration. Consequently, these findings suggest that DXH-GLYS in situ gel could be considered safe.

## Conclusion

Different DXH-GLYS formulations were developed and optimized using the Box–Behnken design. The prepared GLYS could incorporate DXH with high EE%, appropriate vesicle size, and higher permeation ability for DXH across the rectal mucosa. According to the PK study, the optimized DXH-GLYS in situ gel formulation could increase DXH bioavailability by 2.24-folds as compared to oral DXH solution. Thus, the designed DXH-GLYS in situ gel could provide a safe and effective platform for rectal administration of DXH, overwhelming the shortcomings of oral DXH, including low bioavailability and extensive hepatic metabolism. Based on the findings of the PD investigation, rectally administered DXH-GLYS in situ gel exhibited potent antidepressant effectiveness compared to oral DXH administration. Hence, the designed DXH-GLYS in situ gel can potentially achieve clinical benefits in managing depression safely, steadily, and in a long-term manner.

## Supplementary Information

Below is the link to the electronic supplementary material.

Supplementary file1 (DOCX 1280 KB)

## Data Availability

Not applicable.
