# Supplementary material for: Glycerosomal thermosensitive in situ gel of duloxetine HCl as a novel nanoplatform for rectal delivery: in vitro optimization and in vivo appraisal
Source: Drug Deliv Transl Res. 2022 May 27;12(12):3083–103. doi: 10.1007/s13346-022-01172-z (PMC9636110; doi:10.1007/s13346-022-01172-z)
Supplement: Supplementary file 1 — Supplementary file1 (DOCX 1280 KB) [file 13346_2022_1172_MOESM1_ESM.docx]

**Table S1**. Regression analysis results for Y_1_, Y_2_, Y_3_ and Y_4_ responses

| Quadratic model | R^2^ | Adjusted R^2^ | Predicted R^2^ | SD | %CV |
| --- | --- | --- | --- | --- | --- |
| Response Y_1_ | 0.9963 | 0.9898 | 0.9572 | 0.88 | 1.04 |
| Response Y_2_ | 0.9968 | 0.9910 | 0.9616 | 9.44 | 3.50 |
| ResponseY_3_ | 0.9843 | 0.9561 | 0.7683 | 1.95 | 4.12 |
| Response Y_4_ | 0.9895 | 0.9705 | 0.8498 | 12.17 | 2.81 |

**Table S2.** Ex-vivo permeation parameters of DXH-loaded nano-glycerosomes versus DXH solution

| Lag  time  (min) | J_ss_  (μg/cm^2^.h)  (The flux of the drug) | Permeability coefficient  K_p_  (cm/h) |  |
| --- | --- | --- | --- |
| 62.6 ± 3.51 | 25.71 ± 4.21 | 0.0312 ± 0.0021 | F1 |
| 70.43± 2.86 | 23.477 ± 2.33 | 0.0340 ±0.0014 | F2 |
| 95.86 ± 5.98 | 28.50 ± 3.25 | 0.0406 ± 0.0093 | F3 |
| 74.34 ± 1.67 | 24.03 ± 2.34 | 0.0288 ± 0.0038 | F4 |
| 68.47 ± 4.87 | 25.15 ± 1.76 | 0.0307 ± 0.0008 | F5 |
| 21.52 ± 2.97 | 18.44 ± 1.32 | 0.0248 ±0.0001 | F6 |
| 50.86 ± 5.45 | 19.31 ± 1.21 | 0.0208 ± 0.0048 | F7 |
| 41.08± 6.21 | 20.68 ± 2.11 | 0.0237 ± 0.0006 | F8 |
| 27.39 ± 6.54 | 15.62 ± 0.99 | 0.0168 ± 0.0002 | F9 |
| 25.04 ± 5.32 | 14.98 ± 3.11 | 0.0153 ± 0.0094 | F10 |
| 19.56 ± 14.56 | 22.79± 2.99 | 0.0159 ± 0.0004 | F11 |
| 46.95 ± 9.81 | 17.88 ± 4.34 | 0.0205 ± 0.0041 | F12 |
| 55.36 ± 2.38 | 22.30 ± 3.98 | 0.0234 ± 0.0008 | F13 |
| 89.21 ± 1.98 | 26.83 ± 0.87 | 0.0346 ± 0.0002 | F14 |
| 35.21 ± 1.24 | 21.24 ± 1.25 | 0.0238 ± 0.0001 | F15 |
| 28.04 ± 4.78 | 29.74 ± 4.65 | 0.0339 ± 0.0007 | Optimum |
| 20.22 ± 8.91 | 15.37 ± 0.56 | 0.0147 ± 0.0002 | DXH |


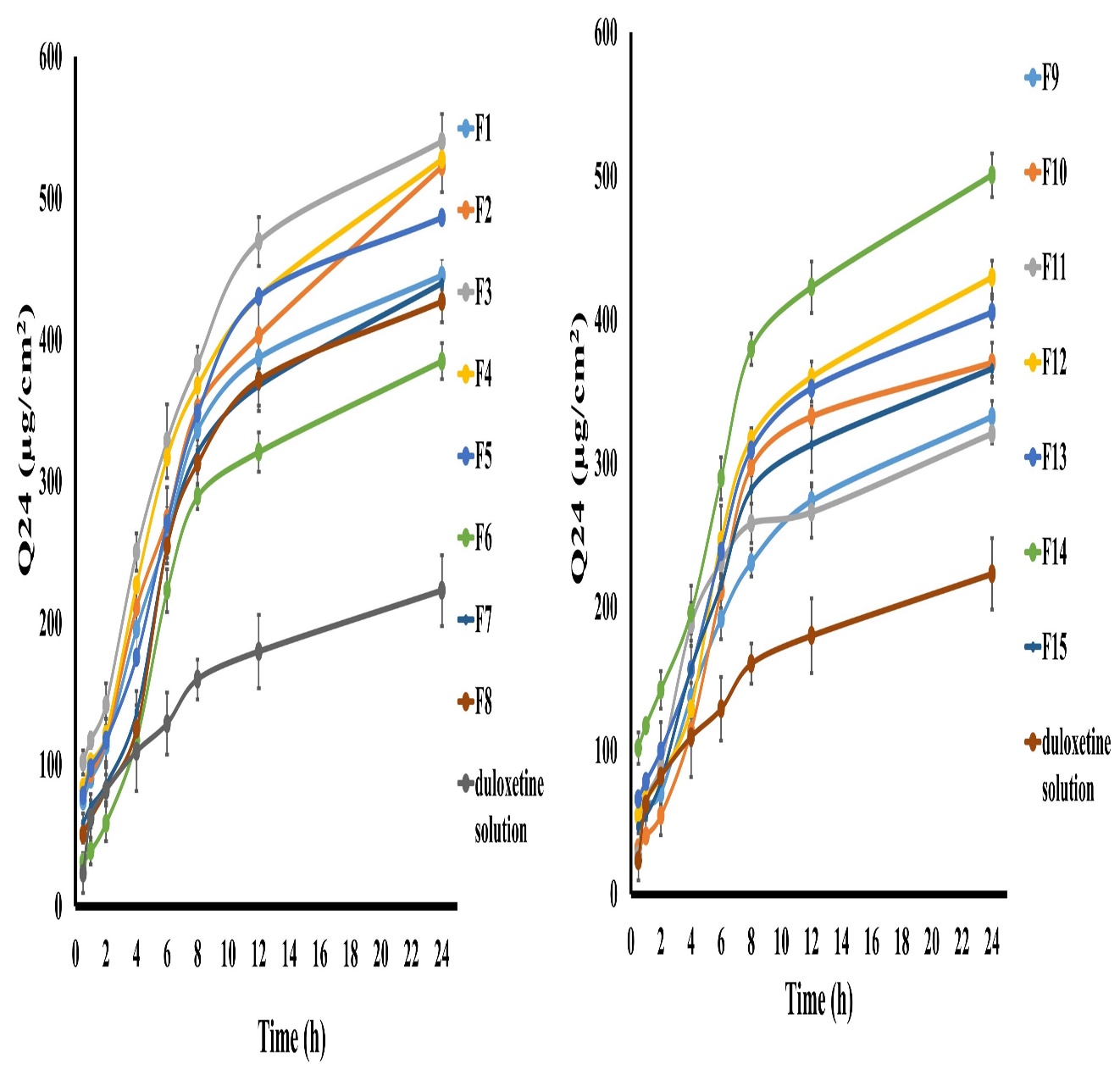


**Figure S1:** Permeation of DXH from different nano-glycerosomal formulations against DXH solution


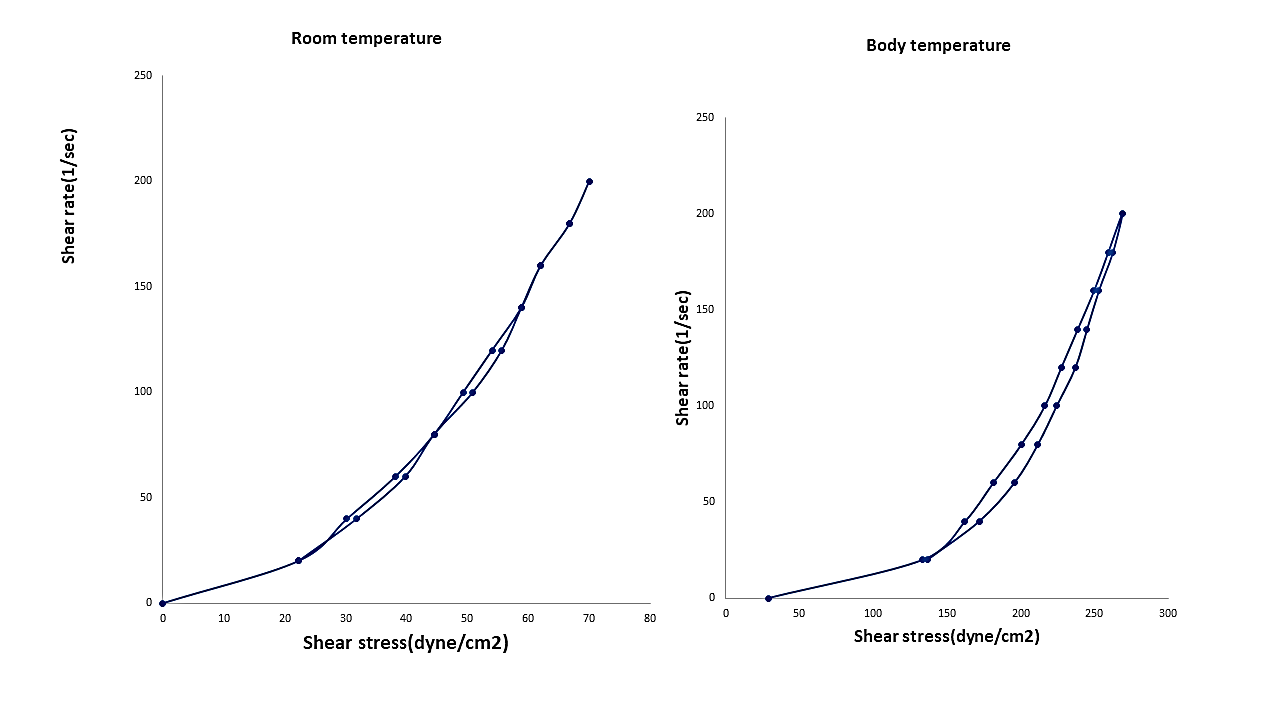


**Figure S2**. Rheogram of the optimized DXH-nanoglycerosomal *in-situ* gel formulation.


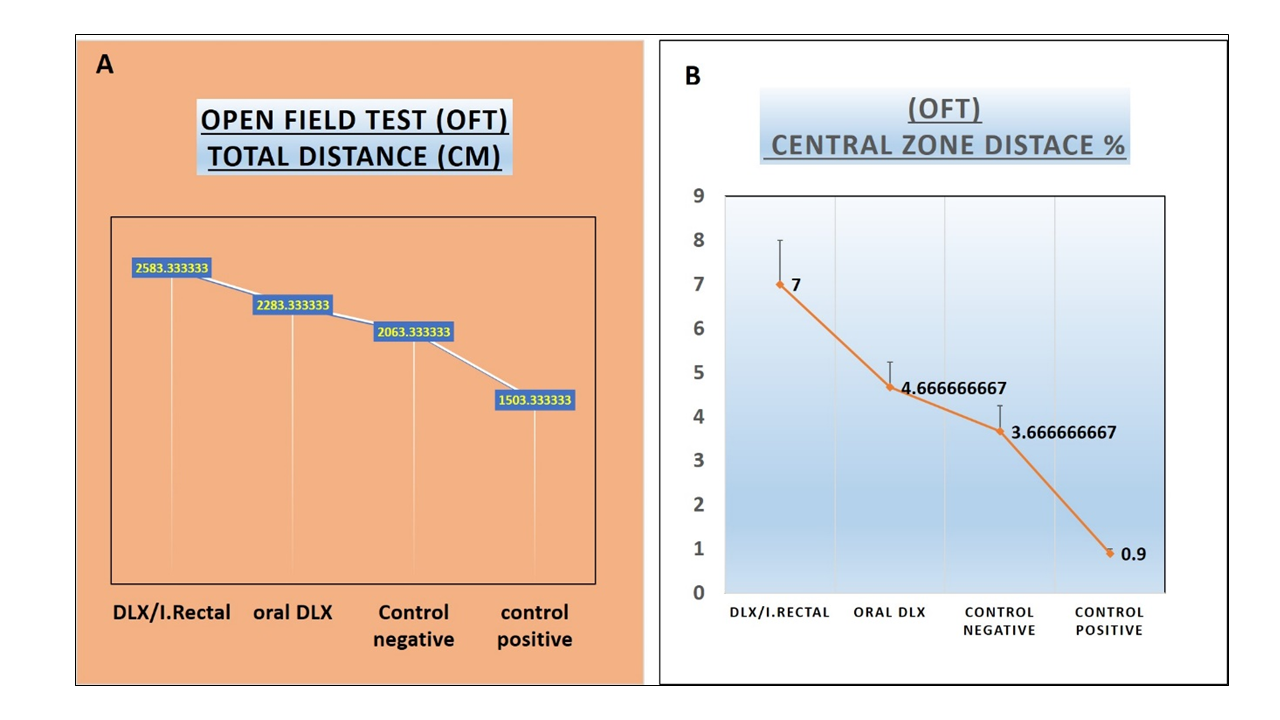


**Figure S3.** The total distance moved in OFT (A), the central distance percentage in OFT (B) were measured in two groups (oral and rectal). Depression exposure decreased the total distance moved and central zone distance percentage in OFT in the control positive group. Results are presented as the mean ± SD*, P< .05*, compared with the control negative normal rats.


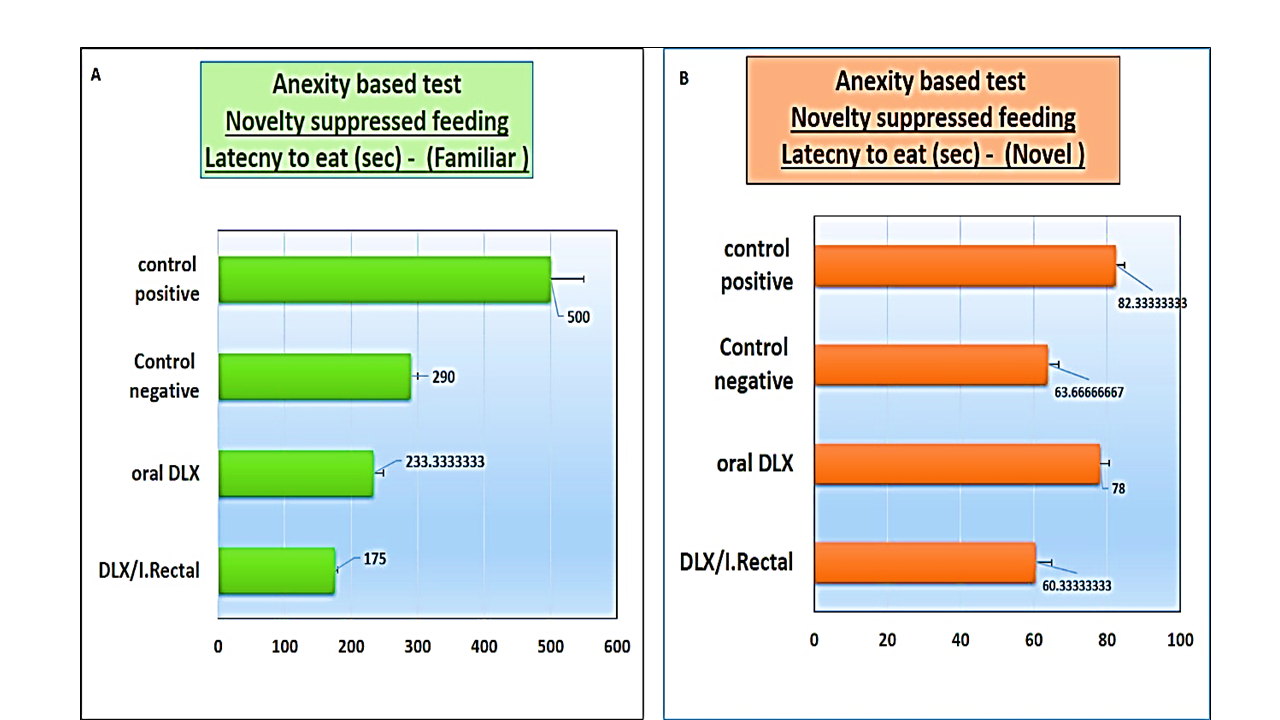


**Figure S4.** Anxiety based or the novelty suppressed feeding test (reduced latency to eat the food in the novel apparatus in rectally administered rats followed by the orally administered group (B)).
